# Sequential substitution of K^+^ bound to Na^+^,K^+^-ATPase visualized by X-ray crystallography

**DOI:** 10.1038/ncomms9004

**Published:** 2015-08-10

**Authors:** Haruo Ogawa, Flemming Cornelius, Ayami Hirata, Chikashi Toyoshima

**Affiliations:** 1 Institute of Molecular and Cellular Biosciences, The University of Tokyo, Bunkyo-ku, Tokyo 113-0032, Japan; 2 Department of Biomedicine, Aarhus University, 8000 Aarhus C, Denmark

## Abstract

Na^+^,K^+^-ATPase transfers three Na^+^ from the cytoplasm into the extracellular medium and two K^+^ in the opposite direction per ATP hydrolysed. The binding and release of Na^+^ and K^+^ are all thought to occur sequentially. Here we demonstrate by X-ray crystallography of the ATPase in E2·MgF_4_^2−^·2K^+^, a state analogous to E2·Pi·2K^+^, combined with isotopic measurements, that the substitution of the two K^+^ with congeners in the extracellular medium indeed occurs at different rates, substantially faster at site II. An analysis of thermal movements of protein atoms in the crystal shows that the M3–M4E helix pair opens and closes the ion pathway leading to the extracellular medium, allowing K^+^ at site II to be substituted first. Taken together, these results indicate that site I K^+^ is the first cation to bind to the empty cation-binding sites after releasing three Na^+^.

## Abstract (web summary)

The Na^+^,K^+^-ATPase moves three Na^+^ ions out of the cell and transfers two K^+^ ions in the opposite direction. Here the authors use X-ray crystallography to look at the substitution of two bound K^+^ with those in the medium and show that it occurs sequentially through a narrow gate.

## Graphical abstract

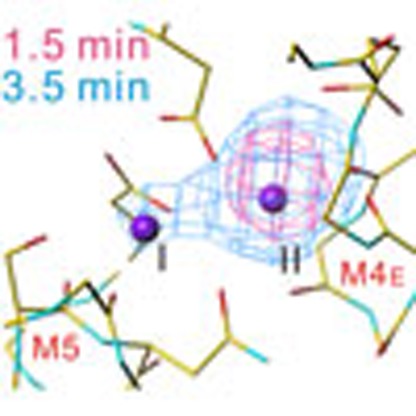

Na^+^,K^+^-ATPase is one of the most important members of the P-type ATPase family. It transports, using the energy liberated by hydrolysis of ATP, three Na^+^ from the cytoplasm to the extracellular side and two K^+^ in the opposite direction in each reaction cycle (for a review, see, for example ref. 1). Na^+^,K^+^-ATPase is the main active transport system responsible for maintaining electrochemical gradients of Na^+^ and K^+^ across the plasma membrane in all animal cells. Such gradients are essential for cells in, for instance, generating action potentials and regulating cell volume. Na^+^,K^+^-ATPase is the target protein of cardiotonic steroids, such as ouabain and digoxin, which have been prescribed for treatment of heart failure for >200 years.

The reaction mechanism of Na^+^,K^+^-ATPase is often described by the Post-Albers scheme^2^,^3^, which includes two major states termed E1 and E2 and phosphorylation of an aspartic acid residue. Transmembrane cation-binding sites in E1 have high affinity for Na^+^ and face the cytoplasm. The binding sites in E2 have low affinity for Na^+^, but high affinity for K^+^ and face the extracellular medium. Active transport of Na^+^ and K^+^ is thought to be achieved by changing accessibility for transporting ions and changing affinity. Binding and release of three Na^+^ and two K^+^ ions all occur sequentially and each step can be distinguished by electrophysiology^4^. There are two occluded states, one for Na^+^ and the other for K^+^, in which bound cations are inaccessible from either side of the membrane^5^. In the reaction scheme shown in Fig. 1, they correspond to E1P[3Na^+^] and E2[2K^+^]. To achieve such occluded states, there must be two gates, one on the cytoplasmic side and the other on the extracellular side, sealing off the transmembrane cation-binding sites. Crystallographic studies of Ca^2+^-ATPase of sarcoplasmic reticulum (SERCA1a)^6^ have provided detailed information on the gating mechanism on the cytoplasmic side from which Ca^2+^ binds, but little on gating on the other side and countertransport of H^+^. It is well established that the cytoplasmic gate corresponds to the M1–M1′ helix and is locked in E1P, but it is obscure what comprises the extracellular gate and how it is locked. This is partly because the countertransported H^+^ is invisible to X-rays. In this regard, Na^+^,K^+^-ATPase has a fundamental advantage over SERCA1a, as it countertransports K^+^ and even its congeners of larger atomic numbers.

At present, the only crystal structures of Na^+^,K^+^-ATPase at better than 3.0-Å resolution with bound K^+^ are those from shark rectal gland in E2·MgF_4_^2−^·2K^+^ with^7^ and without^8^ ouabain. The ATPase in the crystal without ouabain is expected to take a conformation analogous to that in E2·Pi·2K^+^, after hydrolysis of the aspartylphosphate but before the release of Pi, with the extracellular gate in a closed position. The resolution of the crystal structure (PDB ID: 2ZXE) is 2.4  Å^8^, sufficient to show details of the co-ordination of two K^+^ in the high-affinity transmembrane binding sites and of one K^+^ with a regulatory role^9^ in the low affinity cytoplasmic site. We thought that this crystal may be useful in identifying the binding sequence of the two K^+^ from the extracellular side after release of bound Na^+^ into the extracellular medium. Glynn *et al.*^10^ and Forbush^11^ have demonstrated that, in the presence of K^+^ and Pi, substitution occurs very slowly for one of the two bound K^+^ and that the release from one site is blocked by the occupancy of the other. These observations would mean that the (backward) dissociation of two K^+^ into and, therefore, the (forward) binding of two K^+^ from the extracellular side occur in single file. Thus, the K^+^ that dissociates first will be the second K^+^ to bind to the empty cation-binding sites after release of Na^+^. Nevertheless, Morth *et al.*^12^ observed only one phase in the dissociation kinetics of ^86^Rb^+^, a K^+^ congener, with pig kidney ATPase in E2·MgF_4_^2−^·2Rb^+^ and concluded that the ATPase is in an occluded state.

We therefore first demonstrate that only one K^+^ per ATPase is substituted with shark rectal gland ATPase in E2·MgF_4_^2−^·2K^+^. We then proceed to visualize the substitution process utilizing Tl^+^ or Rb^+^ in the medium by X-ray crystallography, as both Tl^+^ and Rb^+^ have larger atomic numbers than K^+^ and measurable anomalous scattering cross-sections. The electron density maps at various incubation times clearly show that the substitution does occur in the ATPase in the crystal lattice and is significantly faster for site II K^+^. Nevertheless, presumably due to slow movements of the gate in the crystal lattice, site I K^+^ is also gradually exchanged. Therefore, we need to clarify to which side and to what extent the gate opens. For this purpose, temperature factors provide useful information. A temperature factor, determined for each atom by X-ray crystallography, shows the size of thermal movements of that particular atom. When all the temperature factors of the atoms constituting a protein are combined, they can be used for characterizing segmental movements^13^. The sidedness of the ion pathway may be determined using ouabain, as it plugs the ion pathway from the extracellular side and will stop substitution via this route^7^,^14^. By combining these strategies, we show how the ion pathway leading to the extracellular medium is gated and in what sequence the counter ions bind to the empty transmembrane cation-binding sites after the three Na^+^ have been released.

## Results

### Substitution kinetics of bound K^+^ by isotopic measurements

**Figure 2:**
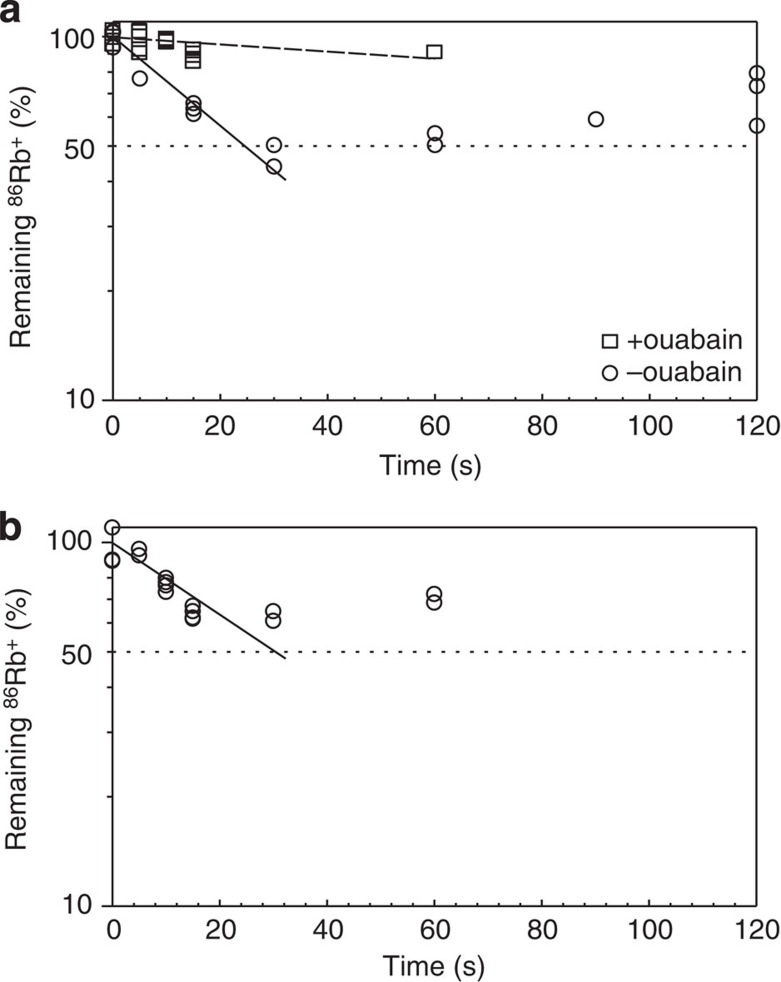
Isotopic measurements of the substitution of bound ^86^Rb^+^ with K^+^ congeners. Substitution of ^86^Rb^+^ in shark rectal gland Na^+^,K^+^-ATPase in E2·MgF_4_^2−^·2Rb^+^ with Rb^+^ (**a**) or Tl^+^ (**b**) at 20 ^o^C. The lines represent single exponential fits to the measurements in the absence of ouabain (circles) up to 30 s with time constants of 14±1 s (**a**) and 17±2 s (**b**), assuming that only half of the bound Rb^+^ is substituted. Broken line and squares in **a** show Rb^+^ substitution with the preformed ATPase–ouabain complex measured after 2 h of incubation. In the normalization of the data, zero is taken at 10%, corresponding to the dilution of ^86^Rb^+^ in the dissociation buffer. Note that substitution ceases within 30 s when approximately half of the initially bound ^86^Rb^+^ has been substituted.

Here the ATPase was fixed first in E2·MgF_4_^2−^·2Rb^+^ by incubating the ATPase in a buffer containing 2 mM RbCl (+^86^Rb^+^), 4 mM NaF and 3 mM MgCl_2_. Substitution of the bound Rb^+^ (+^86^Rb^+^) was then allowed for up to 120 s at room temperature, by incubating the ATPase in a buffer containing 150 mM RbCl or TlNO_3_, and stopped by adding ice-cold sucrose. The time course of radioactivity of ^86^Rb^+^ remaining on the ATPase clearly shows fast and slow (or constant) phases (Fig. 2). Approximately half of the bound ^86^Rb^+^ is substituted within 30 s and no further substitution takes place up to 120 s. Provided that the fast phase is completed at 50% substitution, the plots yield time constants of 14±1 s (mean±s.e. *n*=8) (Rb^+^; Fig. 2a) and 17±2 s (*n*=12) (Tl^+^; Fig. 2b) for the fast phase.

The measurements were repeated in the presence of 10 mM ouabain to determine on which side substitution takes place. It is well established that K^+^ enters into the cavity from the extracellular side and ouabain blocks the binding, presumably by plugging the ion pathway^14^. Indeed, ouabain is located in the cavity open to the extracellular side in the crystal structure of Na^+^,K^+^-ATPase in E2·MgF_4_^2−^·2K^+^ soaked in a buffer containing 10 mM ouabain^7^. Because no substitution is observed when ouabain is present (Fig. 2a) and because MgF_4_^2−^ completely suppresses transition into the E1 state^14^, in which the transmembrane cation-binding sites are exposed to the cytoplasm, it is concluded that the bound K^+^ exchanges with ions (congeners) in the extracellular medium.

### Substitution time course visualized by X-ray crystallography

**Figure 3:**
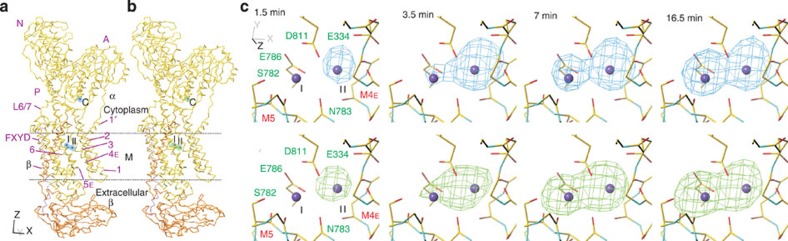
Locations of bound K^+^ and time course of Tl^+^-substitution in the crystal. (**a**,**b**) Whole molecule of shark rectal gland Na^+^,K^+^-ATPase in E2·MgF_4_^2−^·2K^+^ viewed parallel to the membrane; (**c**) transmembrane K^+^-binding sites viewed approximately perpendicular to the membrane from the cytoplasmic side. Superimposed on the atomic models are |*F*_obs_(Tl^+^)|−|*F*_obs_(K^+^)| difference Fourier maps (blue nets in **a** and the upper panel in **c**; contoured at 0.35 e^−^/Å^3^) and anomalous difference Fourier maps (green nets in **b** and the lower panel in **c**; contoured at 0.035 e^−^/Å^3^) at 2.9 Å resolution. The time shown in **c** refers to incubation time before crystals were rapidly frozen in cold nitrogen gas. The maps shown in **a** and **b** are from fully substituted crystals. Colours of the C_α_ traces are: yellow, α-subunit; orange, β-subunit; violet, FXYD10. Purple spheres represent bound K^+^ in the crystal structure. Dotted lines indicate the approximate position of the lipid bilayer (M). See Supplementary Fig. 2. for stereo views of anomalous maps of the full data set.

To visualize the substitution process, crystals of Na^+^,K^+^-ATPase in E2·MgF_4_^2−^·2K^+^ were soaked in a buffer containing 100 mM thallium acetate or rubidium acetate and flash-frozen at various incubation times between 0.75 and 100 min. Diffraction data were collected at wavelengths corresponding to their respective absorption peaks so that both difference (Fig. 3a; Supplementary Fig. 1a) and anomalous (Fig. 3b; Supplementary Fig. 1b) electron density maps could be obtained. The time constants were determined based on 14 (Tl^+^) or 5 (Rb^+^) data sets selected according to resolution (<3.4 Å) and unit cell parameters (Supplementary Table 1). Presented in Fig. 3c are series of difference Fourier (upper panel in Fig. 3c) and anomalous (lower panel; Supplementary Fig. 2 for stereo) maps around the transmembrane binding sites at different soaking times in the Tl^+^-containing buffer. At longer incubation, both types of maps show three distinct peaks per ATPase molecule as expected, exhibiting substitution at all three K^+^ binding sites: two transmembrane (I and II) and one cytoplasmic (C) (Fig. 3a,b and Supplementary Fig. 1). However, at shorter incubation times, substitution of site II K^+^ is much more prominent than that at site I. Essentially, the same maps were obtained for substitution with Rb^+^ (Supplementary Figs 3 and 4).

**Figure 4:**
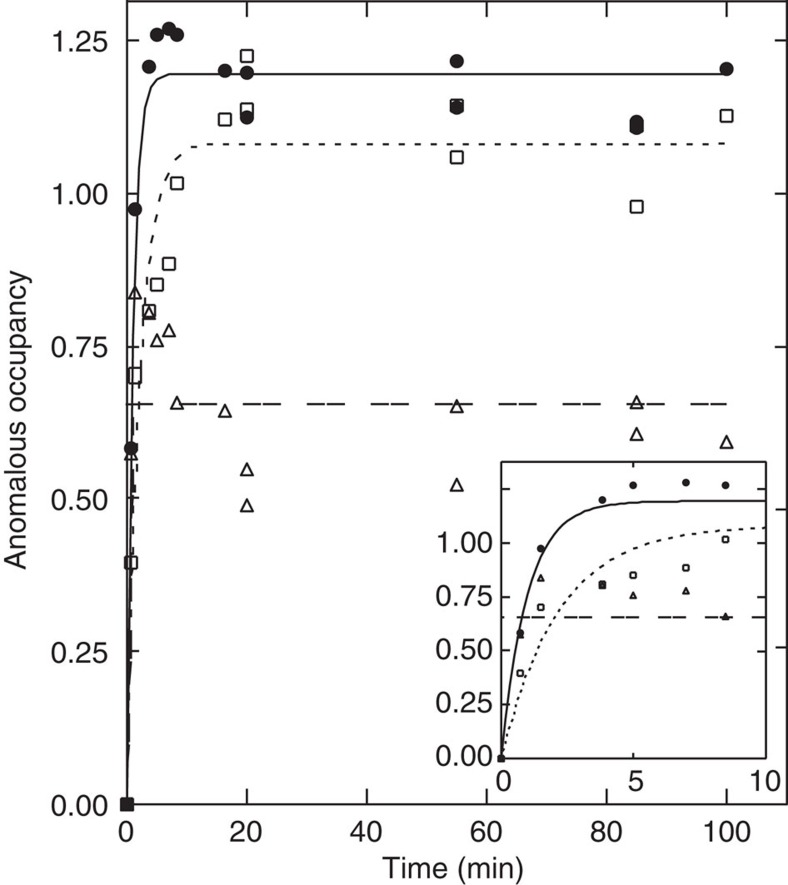
Time course of the change in occupancy of Tl^+^ that has substituted bound K^+^ in the crystal. Open squares, filled circles and open triangles, respectively, represent the anomalous occupancy at sites I, II and C, as determined with CNS^15^. Curves are single exponential fits to the data with time constants of 41 and 105 s. Inset shows the initial part of the same data.

We tried several ways to quantitate the occupancy of Tl^+^ (or Rb^+^) in each site, and found that anomalous occupancy as determined with CNS^15^ gave the most consistent results (Fig. 4). The plots of anomalous occupancy against soaking time thus obtained are well fitted with single exponentials. They yield time constants of 41 s for substitution of site II K^+^ with Tl^+^ and 105 s for site I K^+^. Substitution of site C K^+^ appears too fast to be measured in this way, as the anomalous occupancy shows no change (but at ∼60% of those at the transmembrane sites when the same temperature factor is assumed; Fig. 4).

### Temperature factor analysis

**Figure 5:**
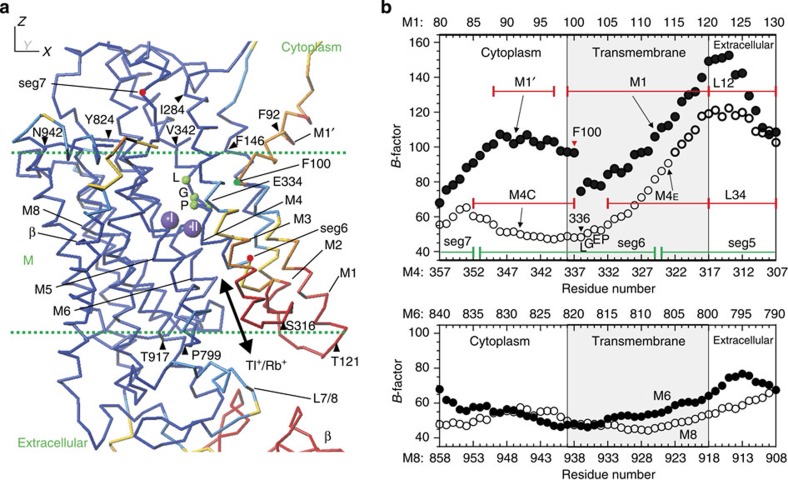
Distribution of temperature factors around the transmembrane K^+^ binding sites. (**a**) A C_α_ trace of the atomic model at 2.4 Å resolution (PDB ID: 2ZXE) after TLS refinement^13^ with 14 segments determined with TLSMD^16^, coloured according to the temperature factors. The colour changes from dark blue (temperature factor of 65 or less), blue (65–75), light blue (75–85), yellow (85–95), orange (95–105), to red (105 or higher). Purple spheres represent bound K^+^. (**b**) Plots of the temperature factor for the C_α_-atoms of the residues in segments that show large thermal movements (M1′-M1 and M4; upper panel) and small movements (lower panel; M6 and M8). M4, M6 and M8 contain residues that coordinate K^+^. E334 in the PEGL motif (small green spheres in **a**) on M4 is considered to be a gating residue on the cytoplasmic side. Residue names with triangles in **a** refer to the ends of segments (specified in **b**) that undergo collective thermal movements.

Thus, substitution occurs substantially more slowly at site I than at site II, but the measurements do not have enough resolution to determine if the substitution occurs sequentially. Additional information is needed to determine how the bound ions are released into the extracellular medium and how the release is gated. Because gating is governed by thermal fluctuations, temperature (*B*) factors determined for all atoms that constitute the ATPase should contain such information. For instance, if gating is achieved by tilting of a transmembrane helix, then the temperature factors of main chain atoms will be small near the pivot and become progressively larger towards the opposite end of the helix. Conversely, if gating is achieved by translation of a transmembrane helix, the temperature factors will be relatively large and similar along the entire helix. Thus, mapping the temperature factors, which would be reasonably accurate at 2.4 Å resolution (PDB ID: 2ZXE), may allow us to identify gating helices and the type of gating movement (Fig. 5 and Supplementary Fig. 5).

Of the 10 (M1–M10) transmembrane helices in the α subunit, the residues located on the M1–M4 helices have considerably higher temperature factors (∼85 for the C_α_ atoms), particularly near the extracellular surface (Fig. 5), going up to ∼100, which is equivalent to random movements of 1.27 Å in radius. Furthermore, the temperature factors are smallest (∼50) for residues just below the cytoplasmic ends. Such features are not observed with residues on the M5–M10 helices, as the temperature factors are almost constant around 54 (Fig. 5), equivalent to thermal fluctuations of 0.68 Å.

The residues that show the lowest temperature factors on the M1–M4 helices are all hydrophobic (Phe100 (M1), Phe146 (M2), Ile284 (M3), Leu336 (M4); identified in Fig. 5) and clustered near the cytoplasmic surface, making extensive van der Waals contacts with residues on neighbouring transmembrane helices. For instance, Phe100 on M1 contacts with Phe291, Ile292 and Ile295 on M3 and with Val332, Pro333 and Leu337 on M4 (Supplementary Fig. 6). In contrast, no such extensive hydrophobic contacts are found in the extracellular halves of M1–M4. From this we can conclude that, for these helices, large movements are allowed near the extracellular ends but strongly suppressed near the cytoplasmic ends. Thus, the M1–M4 helices appear to move together and work as a kind of shutter to the ion pathway.

### Collective movements of transmembrane helices

**Figure 6:**
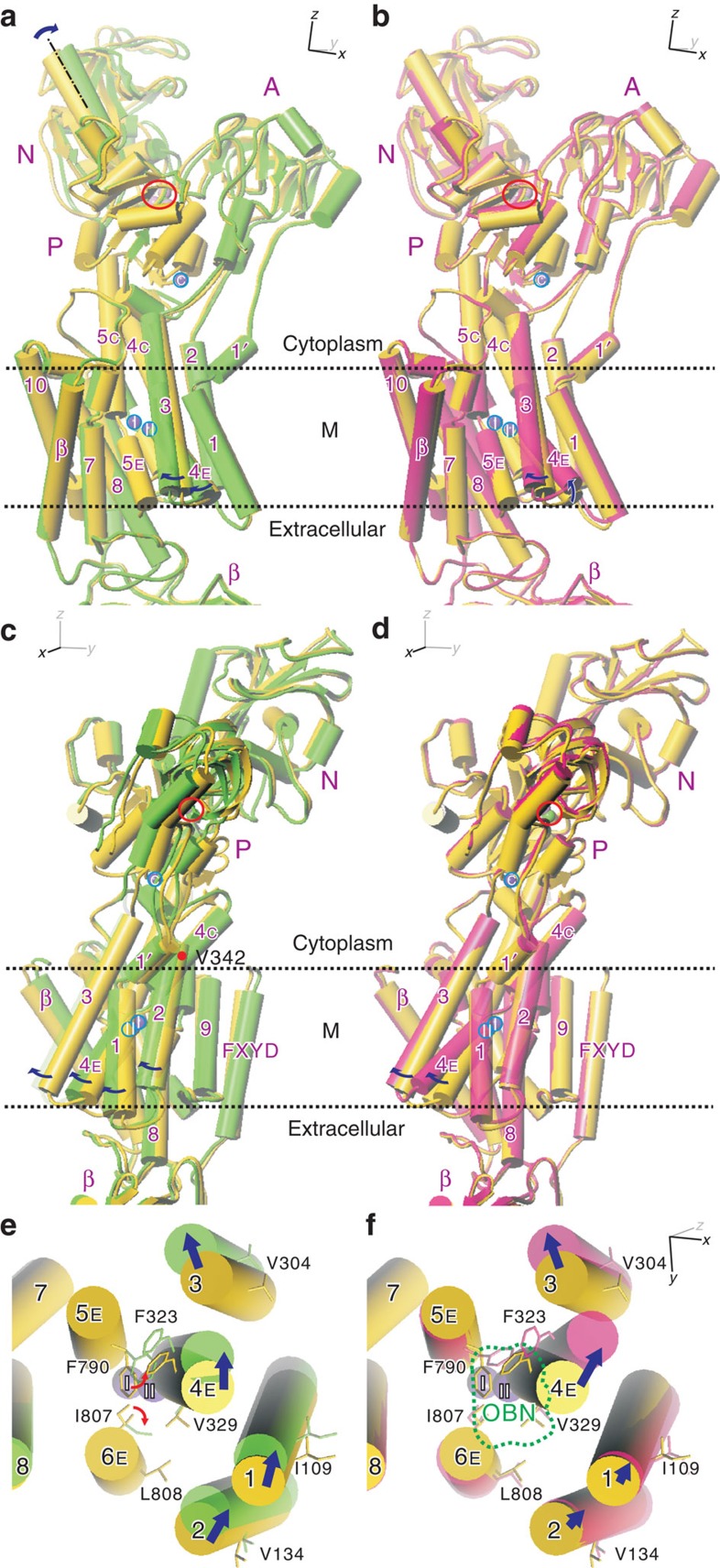
Segmental movements of Na^+^,K^+^-ATPase in the E2·2K^+^·MgF_4_^2-^ crystal. (**a**,**c**,**e**) Superimposition of the atomic models of the ATPase in the ‘average' (yellow; PDB ID: 2ZXE) and ‘open' (light green) conformations. The model in the ‘open' conformation, in which the extracellular pathway to the K^+^-binding sites is most widely opened by thermal movements, was deduced by TLSMD^16^. (**b**,**d**,**f**) Superimposition of the atomic model of the ATPase in the ‘average' conformation (yellow) and that of an ouabain-bound form (PDB ID: 3A3Y; pink). The ouabain-bound form of Na^+^,K^+^-ATPase was made by soaking the E2·MgF_4_^2−^·2K^+^ crystals in a buffer containing 20 mM ouabain. Front views (**a**,**b**), side views (**c**,**d**) of the whole molecule and bottom views (that is, approximately normal to the membrane from the extracellular side) of the transmembrane region (**e**,**f**). Purple spheres (marked I, II and C) represent bound K^+^, and green ones MgF_4_^2−^ (also circled in red). Arrows indicate the directions of segmental movements (from the ‘average' structure to the ‘open' conformation). Small red arrows in **e** indicate likely conformational changes of the side chains to open the ion pathway. Dotted line in **f** shows the outline of ouabain (OBN), which is removed from the atomic model. See Supplementary Fig. 7 for stereo figures of **e** and **f**.

Mapping of temperature factors is useful for determining those parts of the protein that undergo large thermal movements, but cannot identify direction or extent of the movements. Such information can be obtained by analysing the temperature factors of all atoms of the protein together. Thermal movements of an atom can be separated into two components: one coming from anisotropic collective movements of a segment (for example, an α-helix) on which that particular atom is located and the other from the uniform random movements of that atom^13^. What we would like to know are the sizes and directions of the movements of the gating segment and participating residues. Here we employed TLSMD^16^ and REFMAC^13^ with the atomic model at 2.4 Å resolution (PDB ID: 2ZXE) and found that the optimal result, as judged by the lowest *R*_free_ value, was obtained when the α-subunit was separated into 14 segments (TLS groups). Two TLS segments (seg5 and seg6; Fig. 5b) incorporate the M4 helix. One segment (seg6) takes in the C-terminal 2/3 of M4 (325–353), containing the presumed gating residue Glu334, and another segment (seg5; 278–324) includes the extracellular third with most of M3. For further analyses, atomic models provided by TLSMD^16^ (Supplementary Movies 1 and 2) were fitted to the crystal structure with the segment (675–815) containing M5. The atomic model with the largest opening to the extracellular medium is superimposed onto that of the crystal structure (Fig. 6) to examine the extent to which thermal movements open the ion pathway.

**Figure 7:**
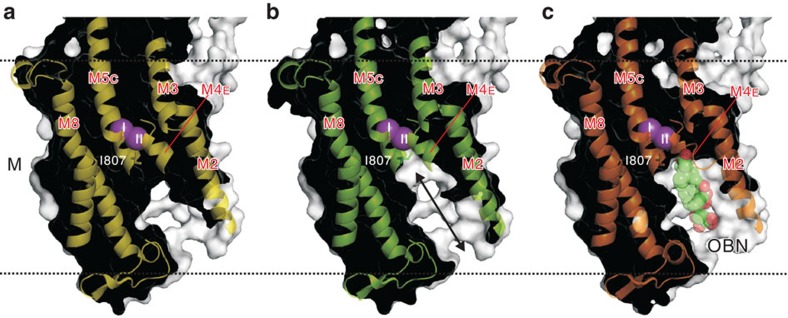
Cross-sections of the transmembrane region of Na^+^,K^+^-ATPase in E2·2K^+^·MgF_4_^2−^. (**a**) The ‘average' conformation (PDB ID: 2ZXE); (**b**) the ‘open' conformation as deduced by TLSMD^16^, showing ion pathway on the extracellular side; (**c**) the ouabain-bound form (PDB ID: 3A3Y). van der Waals surface generated with PyMol. Purple spheres (I and II) represent bound K^+^. Ouabain (OBN) bound to the ATPase with low affinity is shown in space fill. Ile807 on M6 (forming the back wall of the ion pathway in this figure) may change the side chain conformation to open the ion pathway ((**b**), double-headed arrow). Ouabain suppresses such a conformational change of Ile807 (**c**).

**Figure 8:**
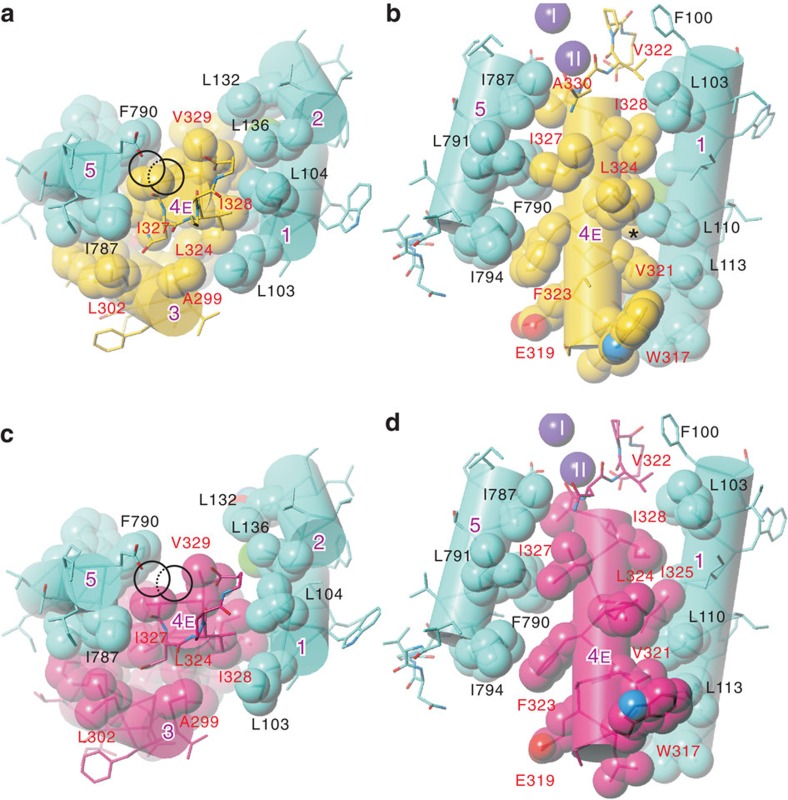
Interface between M4E and the surrounding helices. (**a**,**b**) The ‘average' conformation. (**c**,**d**), The ouabain-bound form. The transmembrane region just below the two K^+^-binding sites (circles) is presented. Viewed from the cytoplasmic side approximately normal (**a**,**c**) or parallel to the membrane (**b**,**d**). Note that M4E is hardly interdigitated with the M1 or M5 helices and that M4E moves together with M3. See Supplementary Figs 9 and 10 for stereo pictures.

Consistent with the temperature factor mapping, M1–M4 helices show large collective thermal movements. Around the K^+^ binding sites, the M4 helix appears to swing within the *yz*-plane by ∼5° with a pivot around Val342 (Fig. 6c) located near the membrane surface, high above the bound K^+^ and the gating residue Glu334. Because of the pivot position, M4E appears to shift by ∼2 Å at Phe323 (Fig. 6e), creating a passage to the extracellular medium below K^+^ at site II (Fig. 7). Such movements of M4E would allow conformational changes of the Ile807 and Phe790 side chains, opening the passage substantially wider (Fig. 6e and Supplementary Fig. 7a). This passage, apparently still too narrow for K^+^ to enter at the cytoplasmic end, becomes sufficiently wide at the extracellular end to accommodate Tl^+^ or Rb^+^ with associated water molecules. As M4E swings within the *yz*-plane, that is, perpendicular to the row of two juxtaposed K^+^ ions, bound K^+^ can only dissociate sequentially from the binding site. Such movement of M4E is possible because the side chains of the residues on M4 that face the M5 and M1 helices are small and only slightly interdigitated with those on M5 or M1 (Fig. 8). It should be noted that the direction of the movement of M4E is not restricted by crystal packing, as there is sufficient space to accommodate movements of M1–M4 as much as 20 Å in the M4–M1 direction (Supplementary Fig. 8a).

These movements of the transmembrane helices are reminiscent of those accompanying the binding of ouabain to Na^+^,K^+^-ATPase (Fig. 6b,d,f). The binding cavity for ouabain is created below site II K^+^, primarily by inclination of M4E (Fig. 6f, Supplementary Fig. 7b). The direction of the movement is similar to that predicted by the TLS analysis but the extent is even larger and accompanies changes in side chain conformations of Leu104 and 110 (M1) and Ile325, 327 and 328 (M4E; Fig. 8; Supplementary Figs 9 and 10). Here M4E appears to incline by 8° (Fig. 6d). In this ouabain-stabilized configuration of the transmembrane helices, the Ile807 side chain cannot change conformation, as it is fixed by ouabain itself (Fig. 7c). Thus, there is no space for K^+^ to get out to the extracellular medium, confirming the suppression of K^+^ substitution as measured by isotopic exchange (Fig. 2a).

Similar collective movements could also be predicted by normal mode analysis of the atomic model (for a recent review, for example, ref. 17). In a simplest form of this approach, a protein is regarded to be consisting of atoms connected by an elastic network and vibrating around local energy minima. The largest principal component involving transmembrane helices obtained by normal mode analyses^18^,^19^ (Supplementary Fig. 7c) indeed shows the movements consistent with the TLS analysis (Fig. 6e, Supplementary Fig. 7a).

## Discussion

Using X-ray crystallography, we have been able to visualize the sequential substitution of two K^+^ bound to Na^+^,K^+^-ATPase in the E2·Pi state, and thereby shown that the K^+^ at site II is the first to be substituted. This sequential substitution takes place because M4E, acting as the gate, thermally fluctuates (swings) to open an ion pathway that connects the transmembrane K^+^-binding sites and extracellular medium.

Half substitution of maximally bound K^+^ in the presence of Pi and Mg^2+^ is a well-established observation (reviewed for instance in ref. 5). For example, Glynn and Richards^10^ measured substitution of ^86^Rb^+^ and ^42^K^+^ bound to dog kidney Na^+^,K^+^-ATPase using ion-exchange resin and found that half of the maximal ^86^Rb^+^ remains bound. Forbush^11^ measured the substitution kinetics of ^86^Rb^+^ bound to pig renal ATPase by rapid filtration, again in the presence of Pi and Mg^2+^. He observed clear fast and slow phases (time constants of 0.1 and 3 s for Rb^+^) and proposed a ‘flickering-gate' model. In this model, two K^+^ ions are juxtaposed in a narrow cavity when bound and are released in single file through a narrow gate. The dwell time of the opening is very short and allows only the K^+^ ion located closer to the gate to be substituted.

A problem in these experiments is that it is ambiguous in which state(s) the substitution takes place, as several reaction intermediates coexist in the presence of Pi, Mg^2+^ and K^+^. Here we used MgF_4_^2−^ as a phosphate analogue that fixes the ATPase in the product state (E2·Pi) of the hydrolysis reaction of the aspartylphosphate and demonstrate that the substitution indeed occurs in two distinct phases in this state. The time constant for the fast phase obtained here by isotopic exchange (∼15 s) is considerably slower than the reported value (∼0.1 s)^11^, indicating that substitution is highly restricted in this fixed state. This slower substitution is related to the fixation of the A-domain. In the normal reaction cycle, the opening and closure of the extracellular gate is directly related to the movement (a 110° rotation) of the A-domain, which is transmitted to the M1–M4 helices. Therefore, the freedom of the A-domain has a critical impact on the substitution kinetics. In E2·MgF_4_^2−^·2K^+^, the A-domain is fixed to the other cytoplasmic domains by a hydrogen bond (between Glu221 (A) in the TGES motif and Thr378 (P)) and a salt bridge (between Glu223 (A) and Arg 551 (N)). But a hydrogen bond between (protonated) Glu221 carboxyl and a fluorine atom in MgF_4_^2−^ appears to be the most significant one, as reactivation of the E2·MgF_4_^2−^·2K^+^·complex (that is, release of MgF_4_^2−^) takes a very long time (only 16% recovered at 1 h) even in the presence of 150 mM Na^+^ (ref. 14). Hence, just as expected, substitution of bound K^+^ occurs much more slowly with the ATPase in E2·MgF_4_^2−^·2K^+^ than that in the presence of Pi, Mg^2+^ and K^+^.

The X-ray crystal structures and measurements of substitution kinetics described here provide strong support, though qualitative, for the ‘flickering-gate' model^11^. Such sequential substitution occurs because the gate, the M4E helix, moves in a plane (that is, the *yz*-plane) approximately perpendicular to the row of two K^+^ (Fig. 6e), allowing only one K^+^ at a time to enter the pathway leading to the extracellular medium. Such movements of M4E are possible because the residues on the M1–M4 and M4–M5 interfaces are not highly interdigitated (Fig. 8). Movements in the perpendicular direction, which might allow substitution of two K^+^ at once, would be severely limited, as the M1–M2 V-shaped structure and, accordingly, the tightly fixed A-domain also have to be moved.

Thus, the extracellular gate is located close to the bound K^+^ (Fig. 7), as M4E is an important part of the K^+^ binding site II (ref. 8), and is likely to be affected by mutations of residues on M1. In fact, Leu99 on the M1 helix of rat α1 Na^+^,K^+^-ATPase (corresponding to Leu104 of shark rectal gland Na^+^,K^+^-ATPase described here) is critical in an interaction with K^+^ (ref. 20). The Leu99Ala mutation reduces the K^+^ affinity in activating the E2P→E2 transition, whereas the Leu99Phe mutation increases the rate of K^+^ deocclusion. Such results are understandable as this residue is located very close to the top (cytoplasmic end) of M4E and contacts Ile328 (Fig. 8), the pivoting residue of M4E.

Quantitatively, however, time constants obtained by X-ray and isotopic exchange are different and distinction between the two phases is not so evident in the X-ray measurements (only 2.5 times difference, not 30 times as observed by Forbush^11^). Tight crystal packing around the A-domain (Supplementary Fig. 8) would limit the movement of the A-domain even further, and thereby that of M4E. It is likely that the highly viscous medium used in the X-ray measurements (40% (wt/vol) PEG 3,000 and 25% (wt/vol) glycerol) has slowed down the movements of M4E, giving enough time for site I K^+^ to escape from the binding site. It is well known that a viscous medium suppresses rapid thermal movements of protein^21^. Thus, the movement of M4E in the X-ray measurements is expected to be highly suppressed and slowed down, resulting in slower substitution and less distinct two phases than those in isotopic measurements.

Is E2·MgF_4_^2−^·2K^+^ an occluded state? To answer this question, a crystal structure of the proper occluded state may be necessary. As no crystal structure is available for E2·[2K^+^] of Na^+^,K^+^-ATPase, we can only refer to the corresponding structure of SERCA1a. The lumenal gate of SERCA1a is closed in both E2 and E2·MgF_4_^2–^ (ref. 22), but the A-domain takes different positions and has different interfaces with the other cytoplasmic domains between the two states^23^. In E2, the A-domain has a substantially larger interface with the N-domain than that in E2·MgF_4_^2−^, much better stabilized with seven hydrogen bonds instead of two, including a salt bridge between Asp203 (A) and Arg489 (N)^24^. These hydrogen bonds will strongly restrict the movement of the A-domain and thereby the M4 helix. This is likely to be the mechanism for occlusion in E2, and explains why ATP/ADP is needed for relieving the ATPase from the occluded state^25^. Release of Rb^+^ from E2[2Rb^+^] has a time constant of ∼10 s, and ATP accelerates the process to 50 ms (Rb^+^) and 22 ms (K^+^)^11^. ATP/ADP would destabilize the interface by disrupting the salt bridge^8^, as Arg489 in SERCA1a binds to the α−phosphate of the nucleotide^26^,^27^. Thus, the lock of the extracellular gate appears to be the closure of the cytoplasmic domains. In this sense, it is in a marked contrast with that of the cytoplasmic gate that is locked by suppressing the conformational change of the Glu309 side chain.

Similar to that of SERCA1a, the A–N interface in E2·MgF_4_^2−^·2K^+^ of Na^+^,K^+^-ATPase is small, stabilized by only one salt bridge (Glu223 (A) and Arg 551 (N))^8^. The question of whether the ATPase in this state is occluded, will be answered in the negative, as the A-domain is fixed primarily by MgF_4_^2−^, not by hydrogen bonds between the cytoplasmic domains. That is why ATP/ADP cannot accelerate the substitution process^12^, even though the Arg 551 binds to the nucleotide^28^. Substitution occurs slowly because of MgF_4_^2−^. This situation will clearly be different in the physiological E2·Pi state. As E2·Pi is a transient state and the cytoplasmic headpiece should open spontaneously in the transition from E2·Pi→E2 to change the interfaces between the cytoplasmic domains, the A-domain should not be fixed tightly to other domains in E2·Pi.

In summary, this paper describes the first direct visualization by X-ray crystallography of the substitution process of bound cations in Na^+^,K^+^-ATPase. The two bound K^+^ in the transmembrane binding sites are substituted sequentially with congeners in the extracellular medium, with site II K^+^ exchanging first. This means that site I K^+^ is the first cation to bind to the empty transmembrane cation-binding sites after release of the three Na^+^. An analysis of thermal motions of the ATPase leads us to propose a mechanism for opening and closing the ion pathway on the extracellular side, which is distinctly different from that on the cytoplasmic side.

## Methods

### Preparation of shark Na^+^,K^+^-ATPase

Crude membrane fractions (microsomes) from the rectal gland of shark *Squalus acanthias* were prepared by homogenization followed by washing and isolation by centrifugation in 30 mM histidine, 1 mM EDTA, 0.25 M sucrose, pH 6.8. The microsomal preparation was subsequently purified by sucrose flotation. The microsomes were diluted to 40% sucrose and layered on top of 60% sucrose followed by layers of 35% sucrose and histidine/EDTA buffer without sucrose. After centrifugation at 96,000*g* for 2 h at 4 °C, the bands at the 0/35 and 35/40% interfaces were collected, washed and resuspended in the histidine/EDTA buffer with 0.25 M sucrose. The purified microsomes were incubated with ∼0.15% deoxycholate for 30 min at 20 ^o^C and washed to remove extrinsic proteins and to open sealed vesicles. The membrane fraction was isolated by centrifugation and the pellet was resuspended in histidine/EDTA with 25% glycerol and homogenized followed by centrifugation at 220,000*g* for 1 h at 10 ^°^C. This step is repeated three times^29^. The preparation was suspended in the histidine/EDTA buffer with 25% glycerol and kept at −20 to −80 °C until use. The preparation showed a turnover number of 200 s^−1^ at 37 °C.

### Crystallization and data collection

Solubilized ATPase with octaethyleneglycol mono-*n*-dodecylether (C_12_E_8_) was mixed with a buffer consisting of 100 mM KCl, 4 mM MgCl_2_, 8 mM KF, 5 mM glutathione, 15 mg ml^−1^ C_12_E_8_, 20% (w/v) glycerol and 20 mM Tris buffer/HCl, pH 7.0. The final concentrations of ATPase and phosphatidylcholine were 2.5 and 2.1 mg ml^−1^, respectively. Crystals of Na^+^,K^+^-ATPase in E2·MgF_4_^2−^·2K^+^ were prepared by dialysing the protein solution in a dialysis button against a crystallization buffer consisting of 18% (w/v) polyethyleneglycol 3,000, 25% glycerol (w/v), 5% (v/v) 2-methyl-2,4-pentanediol, 100 mM potassium acetate, 10 mM KCl, 4 mM MgCl_2_, 4 mM KF, 0.1 mM EGTA, 10 mM glutathione, 2 μg ml^−1^ 2,6-di-*t*-butyl-*p*-cresol, 20 mM MES/Tris buffer, pH 7.0, at 25 °C for 1–2 months^8^. After overnight dialysis against a dehydration buffer (crystallization buffer but containing 40% (w/v) polyethylenglycol 3,000), the crystals were soaked for 0.75–100 min in the dehydration buffer containing 100 mM thallium or rubidium acetate instead of KCl. Then, the crystals were flash-frozen in cold nitrogen gas. All diffraction data were collected at BL41XU, SPring-8, at the absorption peak of Tl (0.9785 Å) or Rb (0.8130 Å). Denzo and Scalepack^30^ were used to process diffraction data.

### Calculation

All the diffraction data sets were scaled first to that derived from the atomic model built for E2·MgF_4_^2−^·2K^+^ (PDB accession code: 2ZXE). Only a rigid body refinement (treating the whole molecule as one segment) was performed for each data set. For evaluating the substitution of bound K^+^ with Tl^+^ or Rb^+^, anomalous occupancy was calculated with CNS^15^. The atomic co-ordinates and *B*-factors derived from the rigid body refinement were fixed during the calculation due to limited resolution. Anomalous scattering parameters were refined first, and thereafter the anomalous occupancies for ions at the three binding sites. All calculations were carried out with CNS^15^ and the maps were in absolute scale.

To analyse local and segmental motions in the crystal, the TLS Motion Determination (TLSMD^16^) server ( http://skuld.bmsc.washington.edu/~tlsmd/) was used. The set of TLS groups that gave the lowest *R*_free_ value after refinement using REFMAC^13^ was selected for further analysis. In this case, 14 TLS groups for the α-subunit (amino-acid residues 32–74, 75–100, 101–154, 155–277, 278–324, 325–353, 354–403, 404–462, 463–493, 494–552, 552–674, 675–815, 816–959 and 960–1023) gave the best result. TLS refinement with four segments (that is, three cytoplasmic and one transmembrane domains) had already been carried out in building the original atomic model (PDB ID: 2ZXE)^8^. Yet, the use of these 14 segments instead of the original 4 in the TLS refinement with REFMAC decreased the *R*_work_/*R*_free_ values from 24.6/27.1 to 24.4/26.7% with virtually no change in co-ordinates (root mean-squared deviation=0.088 Å). No further improvement was observed, however, with the iterative use of TLS refinement. Normal mode analyses of the atomic model thus refined were carried out with ANM 2.0 (ref. 18) and ElNémo^19^. TurboFRODO and Pymol (Schrödinger, LLC) were used for making the structural figures.

### Isotopic measurement of the substitution of bound Rb^+^

Binding of Rb^+^ to Na^+^,K^+^-ATPase was performed by incubating∼40 μg of enzyme in a binding buffer containing 2 mM RbCl (+^86^Rb^+^) in the presence of 4 mM NaF, 3 mM MgCl_2_ and 150 mM Tris-HCl, pH 7.0 for 5 min at room temperature. Subsequently, for the substitution of the bound ^86^Rb^+^, the ATPase was incubated in a substitution buffer containing 20 mM RbCl and 150 mM Tris-HCl, pH 7.0 for varying times at 20 °C. NaF and MgCl_2_ were omitted in the substitution buffer, as MgF_4_^2−^ binds so tightly to the ATPase and does not dissociate significantly within 5 min^14^. The reaction was stopped by addition of 1 ml of ice-cold sucrose (200 mM). A 500-μl aliquot of sample was immediately applied to an ice-cold Dowex-50 column (Tris-form) followed by 1.5 ml of ice-cold sucrose (200 mM). The zero-time sample was produced by adding sucrose solution before the dissociation buffer. Dissociation of Rb^+^ from the ATPase by exchanging with Tl^+^ was measured essentially the same as described above, except the dissociation buffer was 20 mM TlNO_3_, 20 mM Tris-HCl, pH 7.0. Substitution of bound Rb^+^ in the presence of ouabain was performed by incubating ∼40 μg of the ATPase for 2 h at room temperature in a buffer containing 2 mM RbCl (+^86^Rb^+^), 4 mM NaF, 3 mM MgCl_2_, and 150 mM Tris-HCl, pH 7.0 in the presence of 10 mM ouabain. Measurement of the dissociation of Rb^+^ from the sample was performed essentially as described above.

## Additional information

**Accession codes**: Atomic co-ordinates and structure factors files have been deposited in the Protein Data Bank under accession codes 5AVQ, 5AVR, 5AVS, 5AVT, 5AVU, 5AVV, 5AVW, 5AVX, 5AVY, 5AVZ, 5AW0, 5AW1, 5AW2, 5AW3, 5AW4, 5AW5, 5AW6, 5AW7, 5AW8 and 5AW9.

**How to cite this article:** Ogawa, H. *et al.* Sequential substitution of K^+^ bound to Na^+,^K^+^-ATPase visualized by X-ray crystallography. *Nat. Commun.* 6:8004 doi: 10.1038/ncomms9004 (2015).

## Supplementary Material

Supplementary Information Supplementary Figures 1-10, Supplementary Table 1 and Supplementary References

Supplementary Movie 1 A movie showing possible thermal movements of Na^+^,K^+^-ATPase in the E2·MgF_4_^2−^·2K^+^ crystal as deduced by TLSMD^1^. Viewed parallel to the membrane plane.

Supplementary Movie 2 A movie showing possible thermal movements of Na^+^,K^+^-ATPase in the E2·MgF_4_^2−^·2K^+^ crystal as deduced by TLSMD^1^. Viewed from the extracellular side perpendicular to the membrane plane.

## Figures and Tables

**Figure 1:**
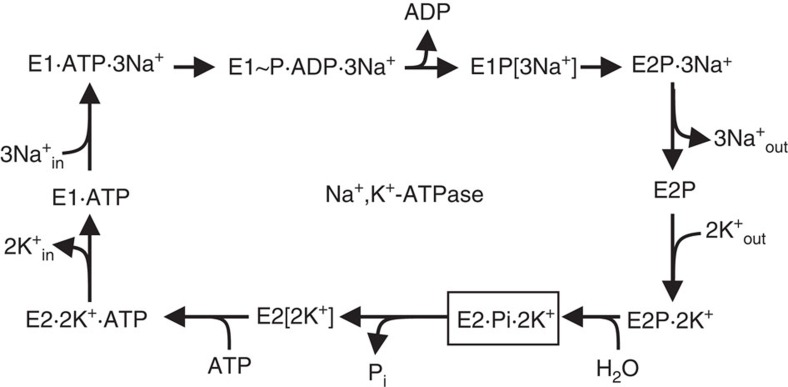
The reaction cycle of Na^+^,K^+^-ATPase according to the classical Post-Albers scheme^2^,^3^. A simplified version showing only the forward direction. The ATPase in the E2· MgF_4_^2−^·2K^+^ crystal is supposed to represent the E2·Pi·2K^+^ state (boxed; the product state of the hydrolysis of the aspartylphosphate). The extracellular gate of the ion pathway is open in the E2P ground state and closed in E2·2K^+^.
