# Supplementary material for: Sequential substitution of K+ bound to Na+,K+-ATPase visualized by X-ray crystallography
Source: Nat Commun. 2015 Aug 10;6:8004. doi: 10.1038/ncomms9004 (PMC4918401; doi:10.1038/ncomms9004)
Supplement: Supplementary Information — Supplementary Figures 1-10, Supplementary Table 1 and Supplementary References [file ncomms9004-s1.pdf]

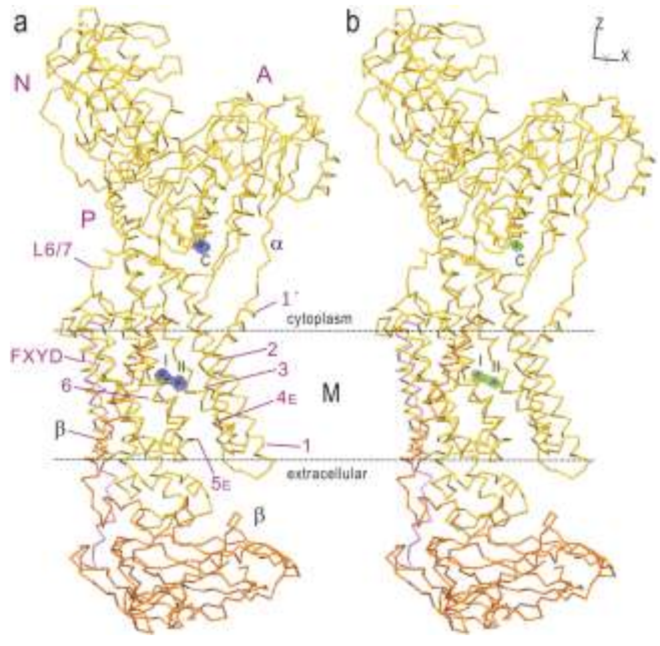

Supplementary Figure 1. **Locations of bound Rb^+ in the crystal of shark rectal gland Na^+,K^+-ATPase in E2·MgF_4^{2-}·2K^+.** (**a**) $|F_{obs}(Rb^+)|$ - $|F_{obs}(K^+)|$ difference Fourier map contoured at 0.15 $e^-/Å^3$ (blue nets); (**b**) anomalous difference Fourier map (cyan nets). Maps were obtained from fully substituted crystals and calculated at 2.9 Å resolution and are superimposed onto the C_α traces (yellow, α-subunit; orange, β-subunit; violet, FXYD10) of the atomic model. Purple spheres represent bound K^+ in the crystal structure (PDB ID: 2ZXE). Dotted lines indicate the approximate position of the lipid bilayer (M).

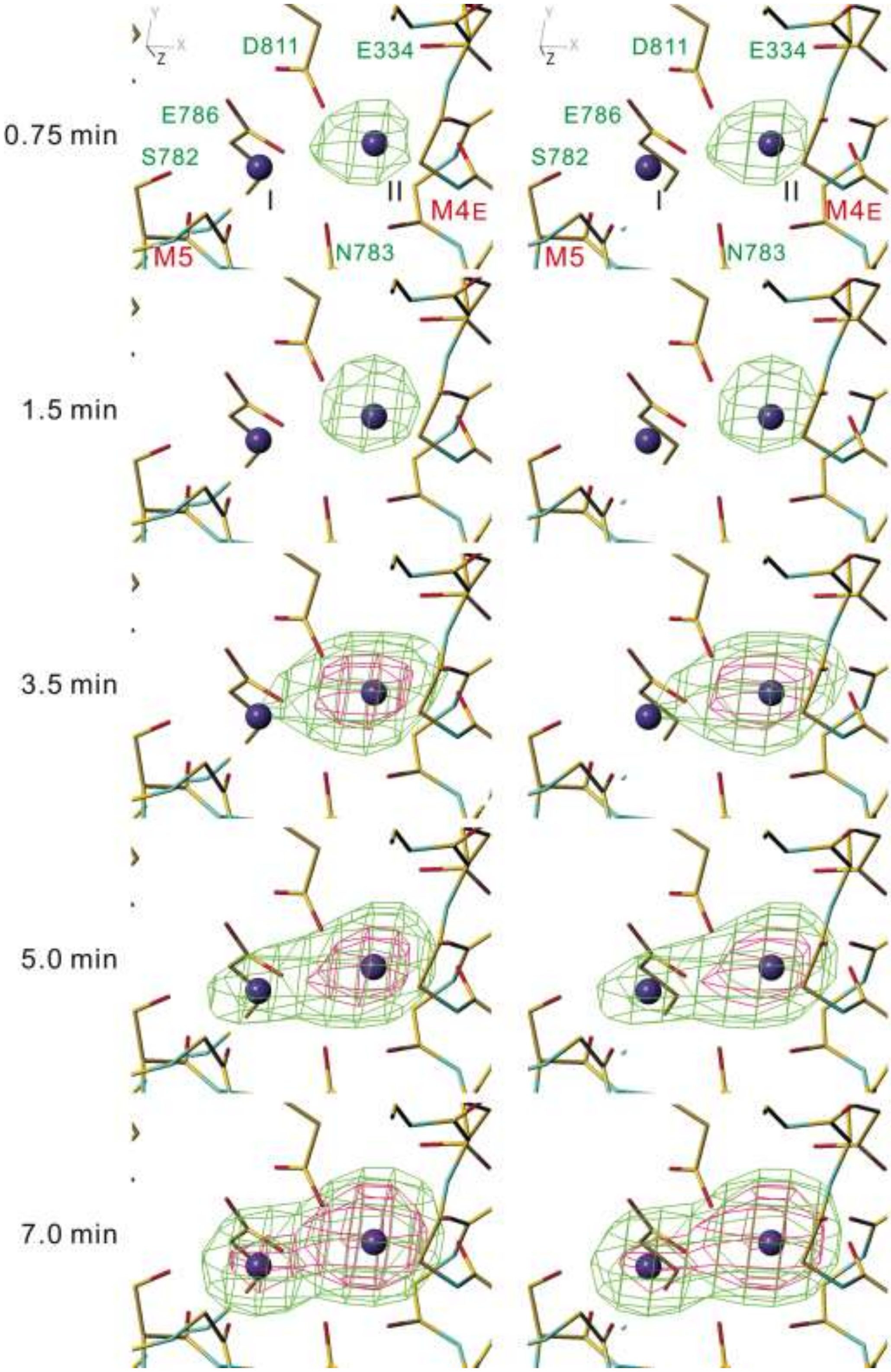

Supplementary Figure 2. Time course of Tl^{+}-substitution of bound K^{+} in the $E2 \cdot MgF_4^{2-} \cdot 2K^{+}$ crystal. Anomalous difference Fourier maps in stereo contoured at 0.035 $e^{-}/Å^{3}$ (green nets) and 0.07 $e^{-}/Å^{3}$ (red nets) using full data sets listed in Table 1. Incubation times in the substitution buffer containing 100 mM Tl^{+} are indicated.

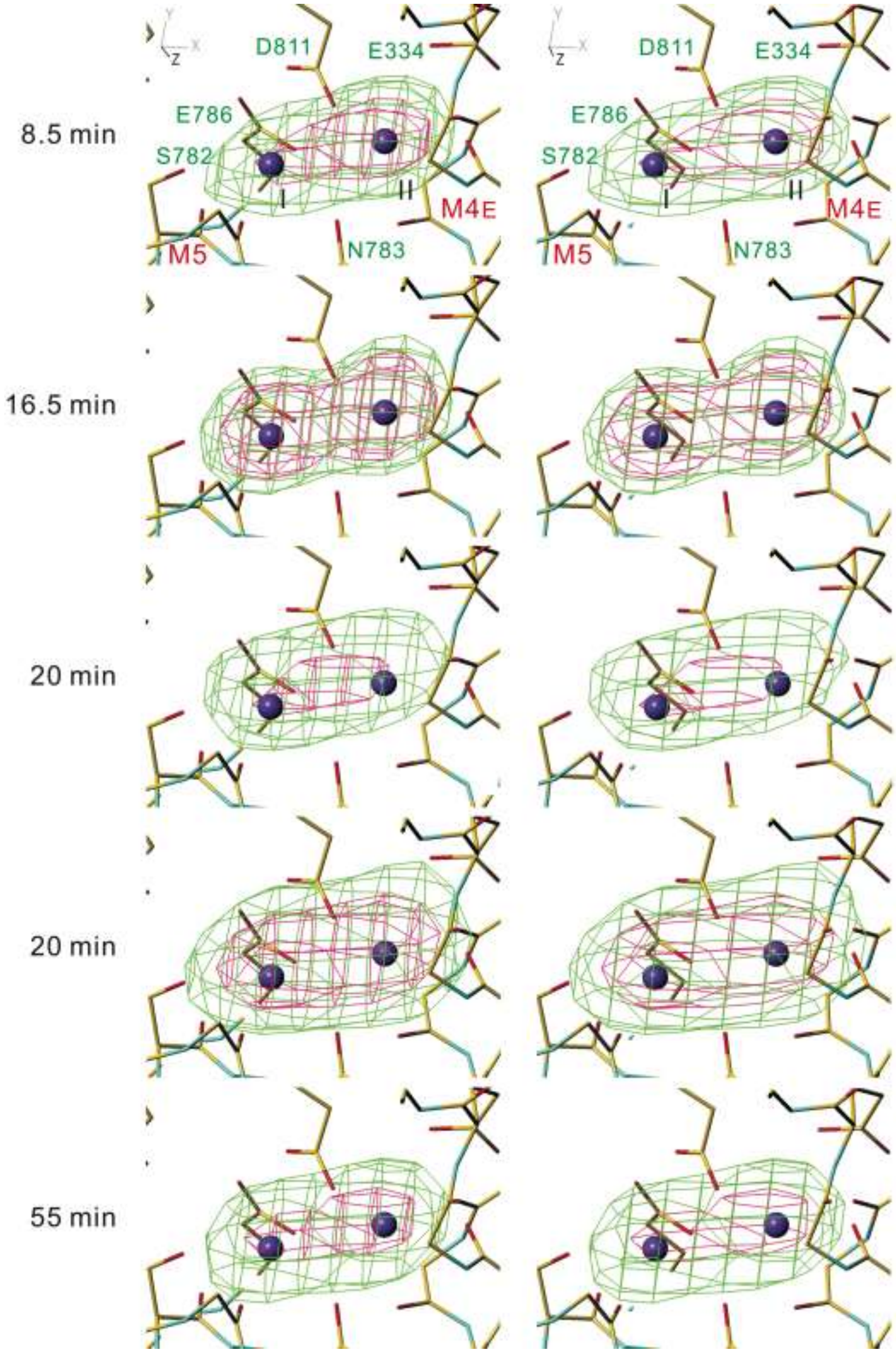

Supplementary Figure 2. (continued)

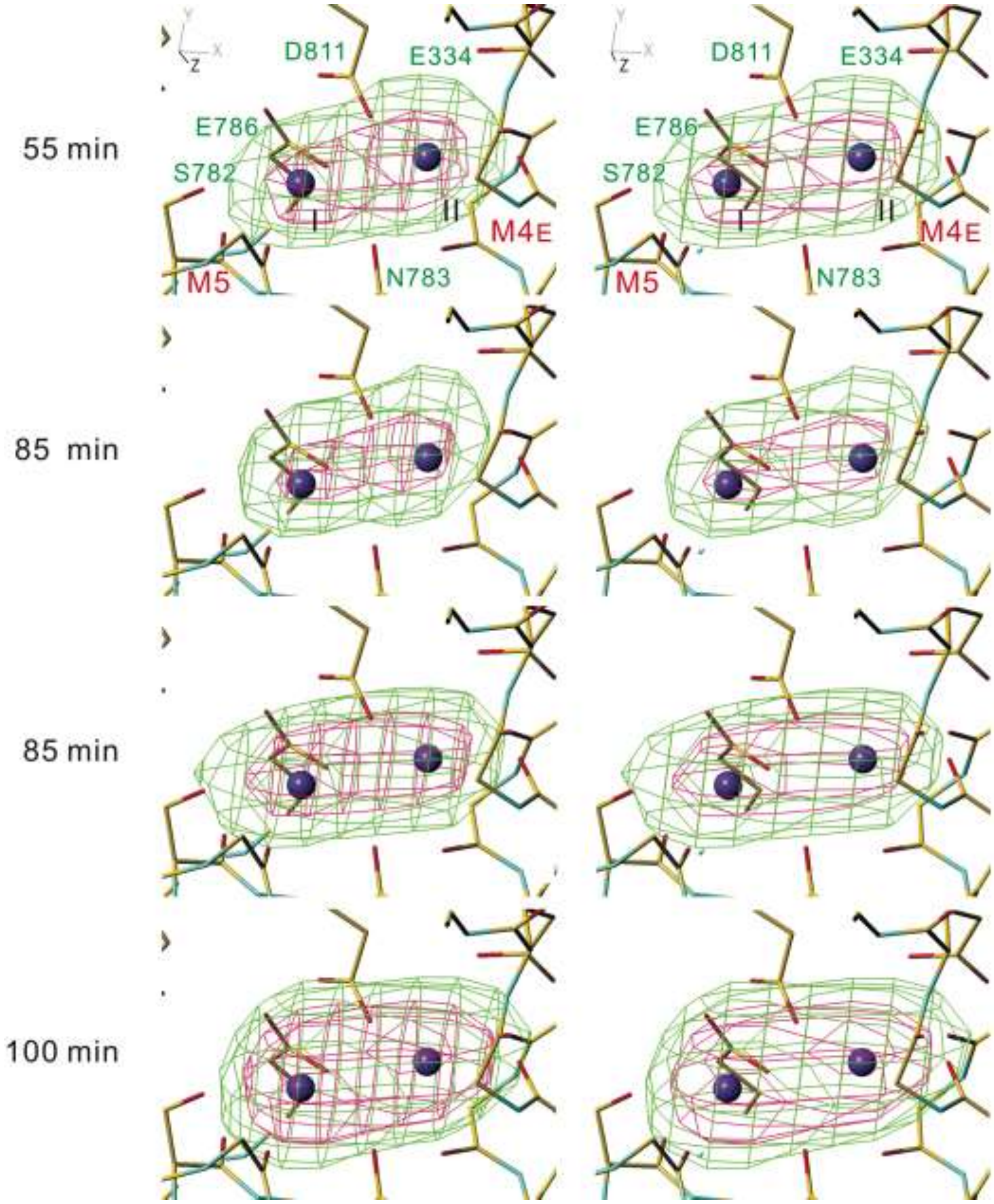

Supplementary Figure 2. (continued)

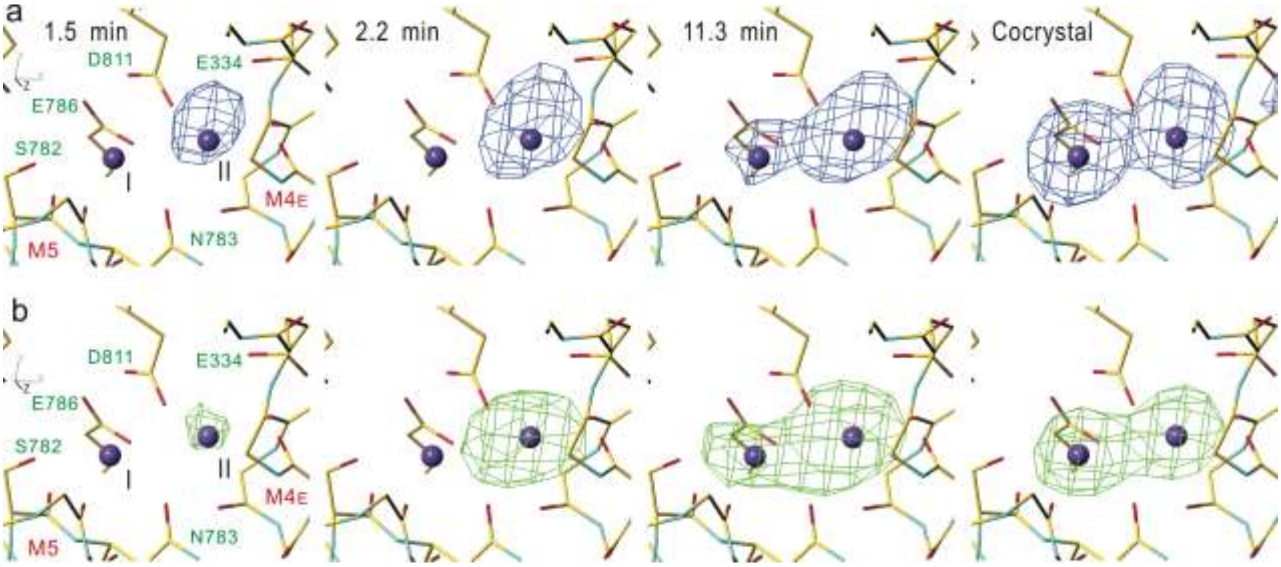

Supplementary Figure 3. **Time course of Rb^+-substitution of bound K^+ in the $E2{\cdot}MgF_4^{2-}{\cdot}2K^+$ crystal.** (**a**) $|F_{obs}(Rb^+)| - |F_{obs}(K^+)|$ difference Fourier maps contoured at 0.15 $e^-/Å^3$ (blue nets); (**b**) anomalous difference maps contoured at 0.03 $e^-/Å^3$ (green nets). Obtained from the crystals flash-frozen at the specified substitution time and calculated at 2.9 Å resolution. Purple spheres represent bound K^+ in the crystal structure. Viewed from the cytoplasmic side nearly perpendicular to the membrane.

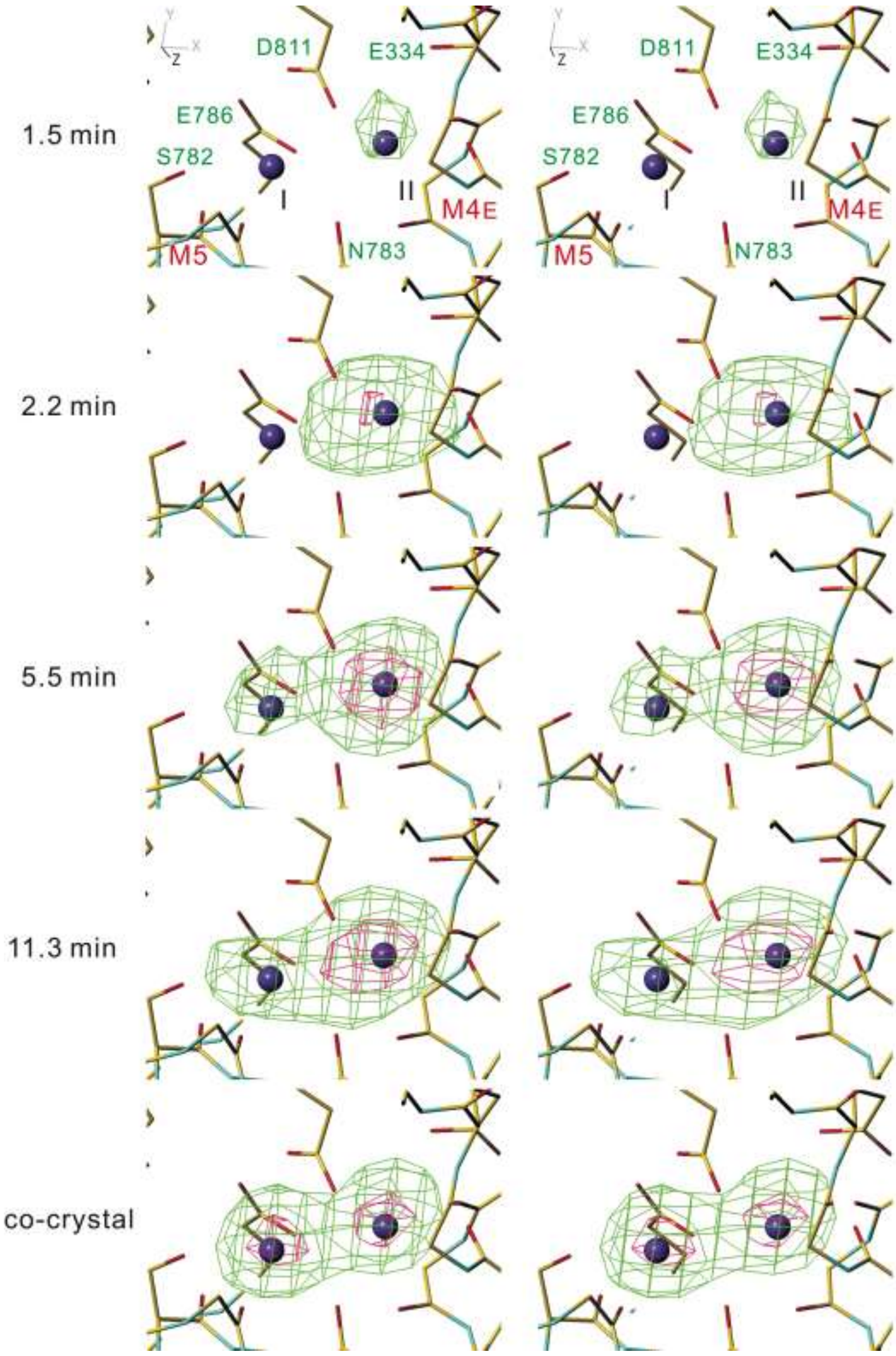

Supplementary Figure 4. Time course of Rb^+-substitution of bound K^+ in the E2·MgF_4^{2-}·$2K^+$ crystal. Anomalous difference Fourier maps in stereo contoured at 0.03 $e^-/Å^3$ (green) and 0.06 $e^-/Å^3$ (red) using full data sets listed in Table 1. Incubation times in the substitution buffer containing 100 mM Rb^+ are indicated. $2|F_{obs}|-|F_{calc}|$ electron density map from the native crystal used as the reference (see Table 1 for the crystallographic data) is shown at 1.6 σ (blue nets)□

2|F_{obs}|-|F_{calc}|

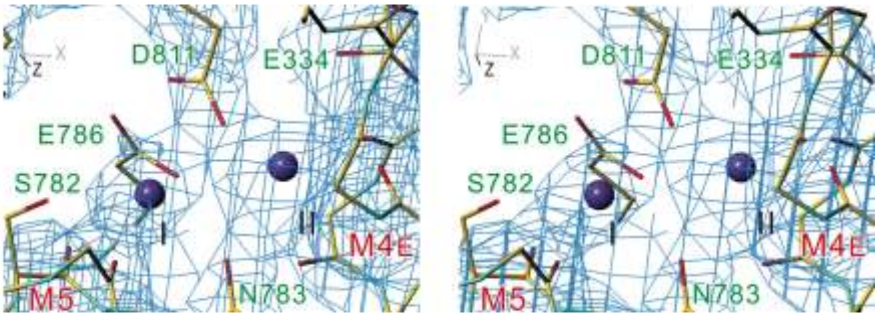

Supplementary Figure 4. (continued)

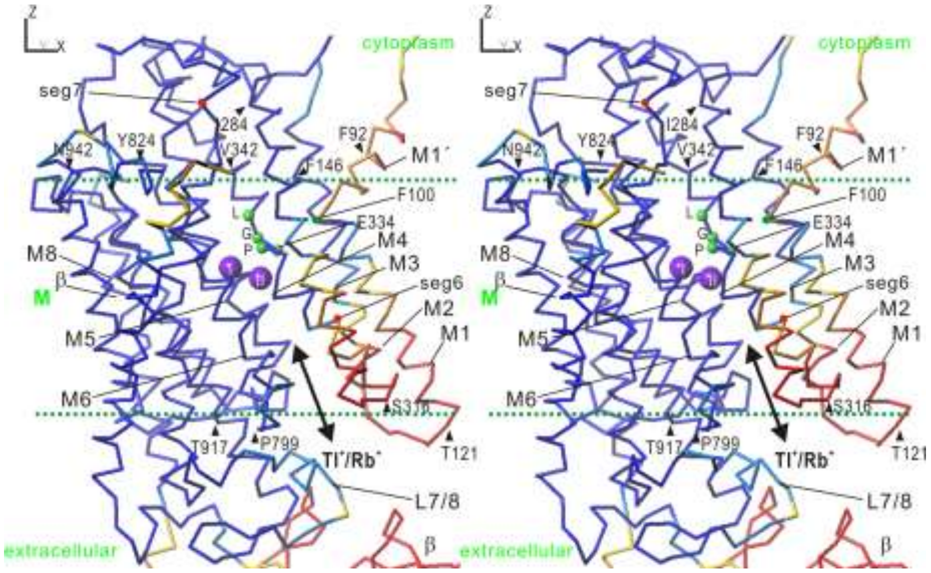

Supplementary Figure 5. **Distribution of temperature factor around the transmembrane K^+ binding sites.** The colour changes from dark blue (temperature factor of 65 or less), blue (65 to 75), light blue (75 to 85), yellow (85 to 95), orange (95 to 105), to red (105 or higher). Purple spheres represent bound K^+. A stereo version of Fig. 5a.

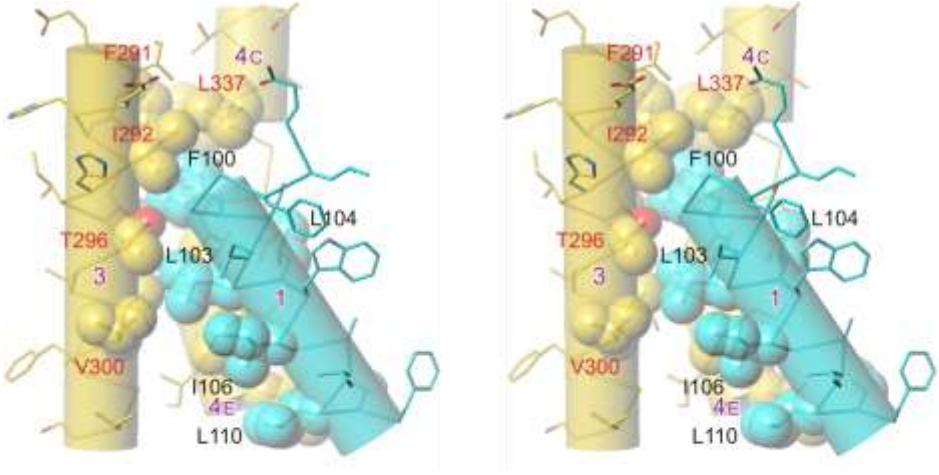

Supplementary Figure 6. **Interface between the M1 and M3 helices.** A stereo picture. Note that the Phe100 side chain is inserted into a pocket formed by hydrophobic residues on M3 and M4 helices, including Phe291 and Ile292 (M3) and Leu337 (M4), suggesting that Phe100 works as a kind of swivel in movements of M1. Note also that M1 and M3 are hardly interdigitated, allowing movements of M1 towards the reader.

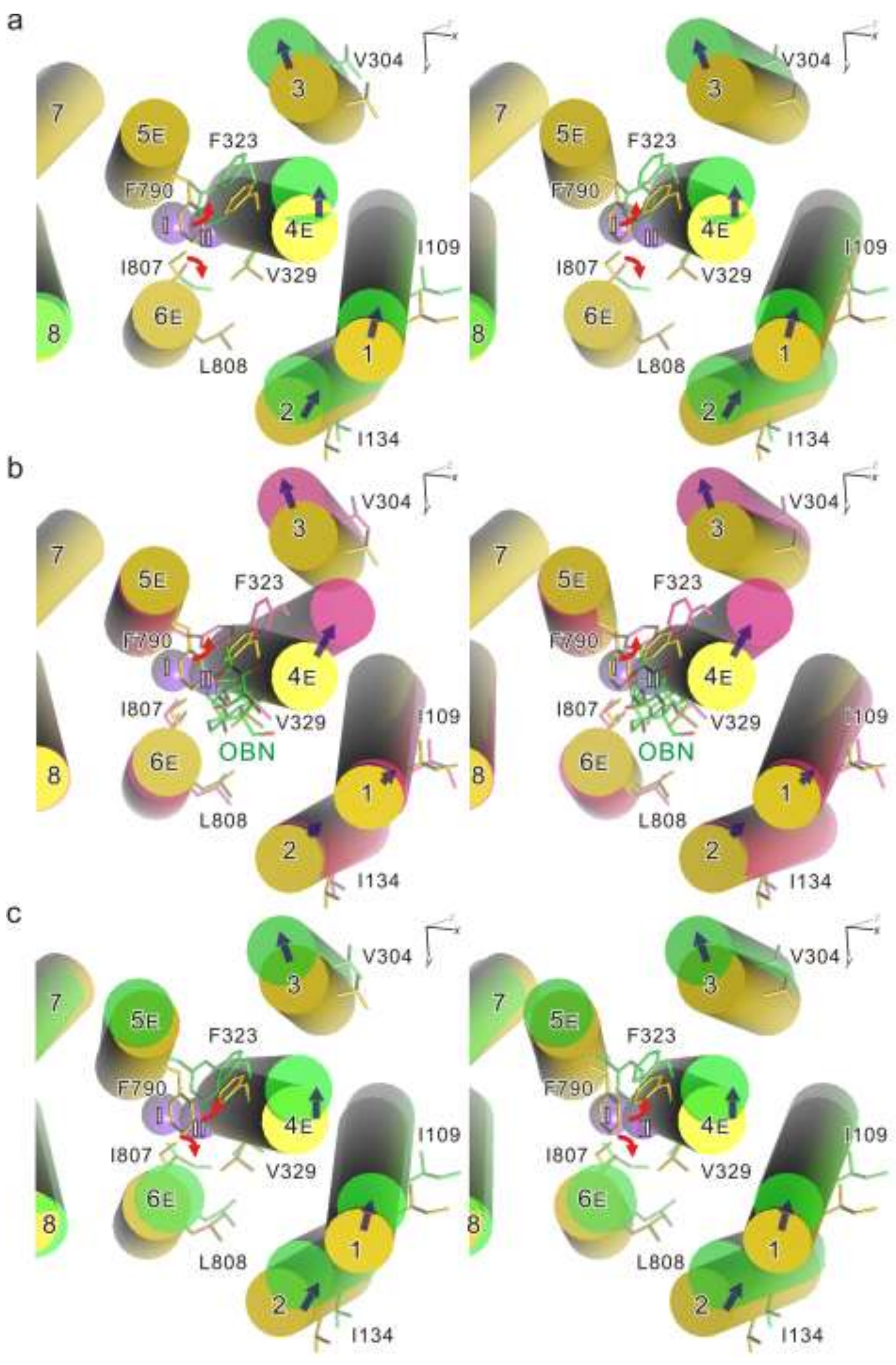

Supplementary Figure 7. **Segmental movements of the transmembrane helices of Na^+,K^+-ATPase in the $E2{\cdot}MgF_4^{2-}{\cdot}2K^+$ crystals.** (**a, c**) Superimposition of the atomic models of the ATPase in the "average" (yellow; PDB ID: 2ZXE) and "open" conformations (green) as deduced by TLSMD[1] (a stereo version of Fig. 6e; **a**) or ANM 2.0 (ref 2, **c**). The "open" conformation in **c** corresponds to the 12th largest component but shows the largest movements in the transmembrane region. It is also the largest in collectivity (0.711)[3]. (**b**) Superimposition of the atomic model of the ATPase in the "average" conformation (yellow) and that of an ouabain (OBN)-bound form (PDB ID: 3A3Y; pink). A stereo version of Fig. 6f. Viewed from the extracellular side approximately perpendicular to the membrane. Small red arrows indicate likely conformational changes of side chains to open the ion pathway. Note that Ile807 cannot change the side chain conformation due to contact with ouabain (**b**).

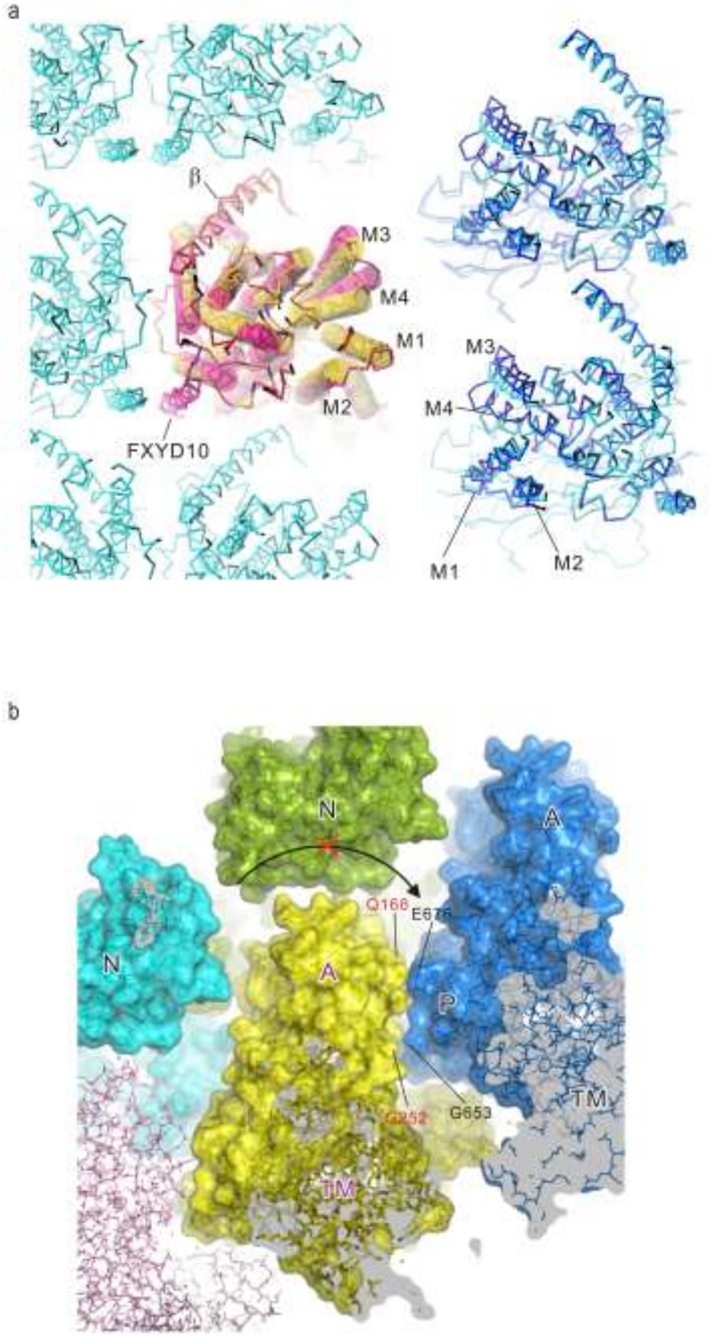

Supplementary Figure 8. **Packing in the E2·MgF$_4^{2-}$·2K$^+$ crystals.** (**a**) Transmembrane region, showing that plenty of space is available for movements of M1-M4 helices away from the M5-M10 helices. (**b**) Cytoplasmic region, showing that the A-domain can hardly move due to contacts with the P-domain of the adjacent molecule. An intermolecular hydrogen bond between Gln168 (A) and Glu676 (P) and a tight crystal contact between Gly252 (A) and Gly653 (P) suppress rotational movements (arrow) of the A-domain. Viewed from the extracellular side. The β-subunit is removed for clarity in both panels, and the transmembrane domain of the α-subunit is also removed in **b**.

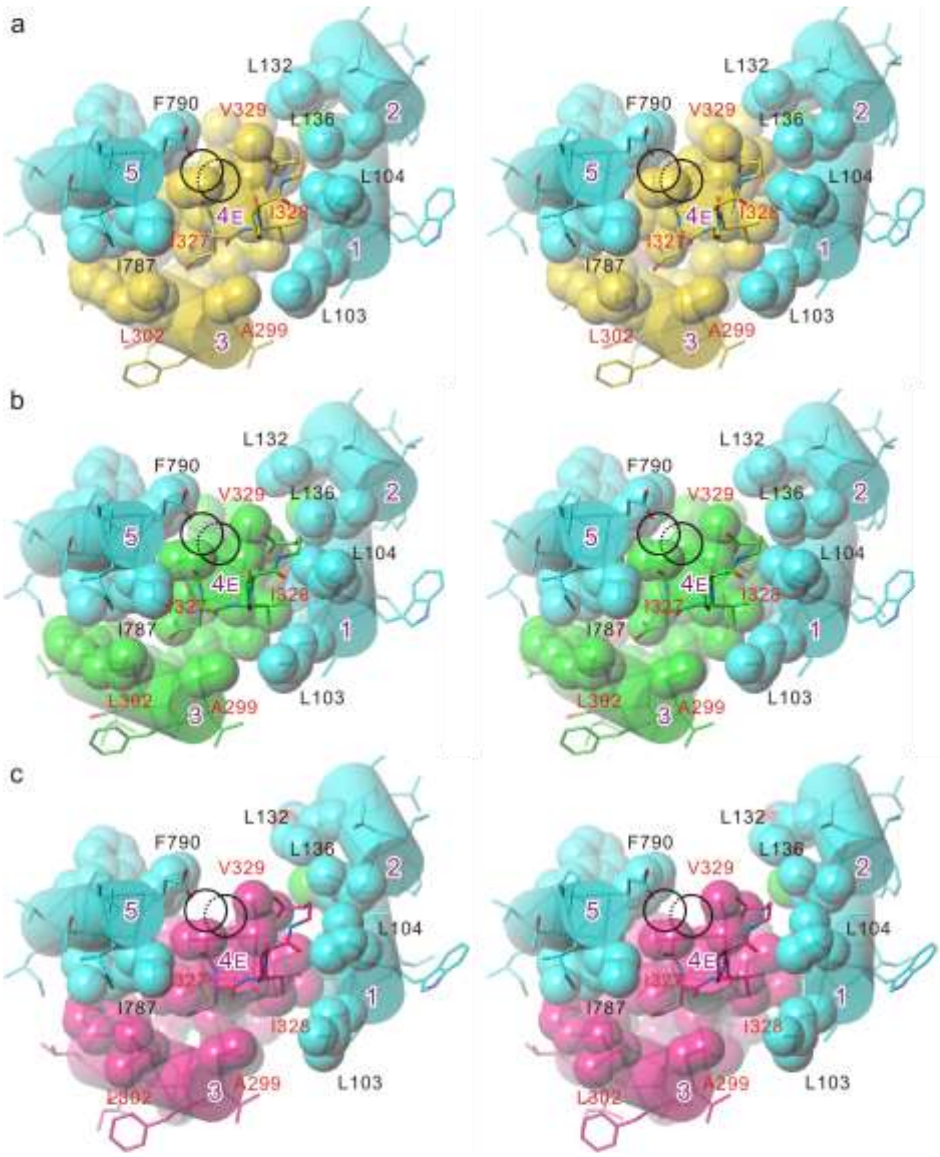

Supplementary Figure 9. **Movements of the extracellular half of the M1-M5 helices of Na^{+},K^{+}-ATPase in the E2·MgF_4^{2-}·$2K^{+}$ crystals.** (**a**) The "average" conformation (PDB ID: 2ZXE); (**b**) thermally "opened" conformation as deduced by TLSMD[1]; (**c**) ouabain bound form (PDB ID: 3A3Y). Side chains of residues on M4E and those facing M4E are in space fill. Black circles show bound K^{+}. Stereo views from the cytoplasmic side approximately perpendicular to the membrane.

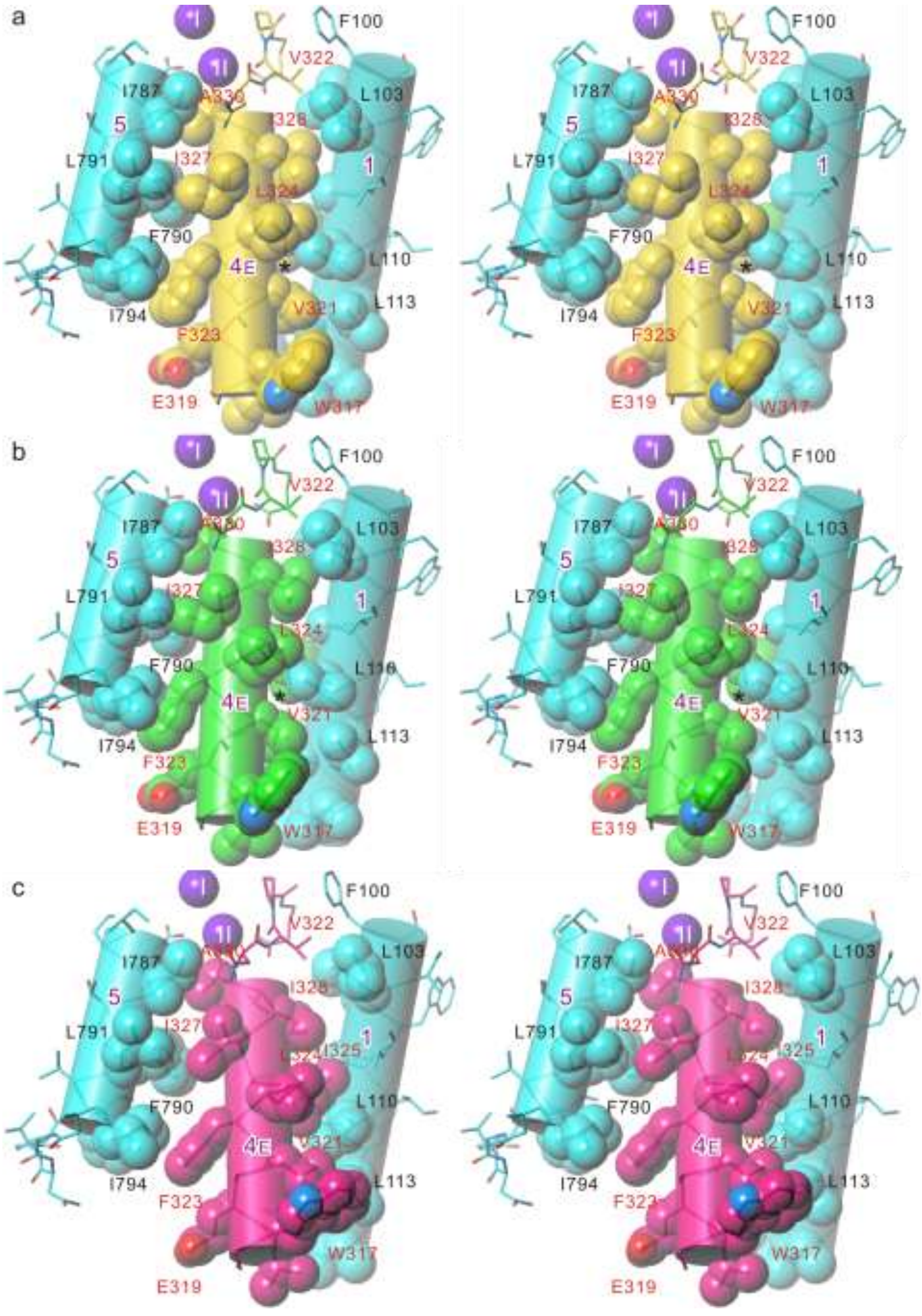

Supplementary Figure 10. Movements of the extracellular half of the M1,M4 and M5 helices of Na^{+},K^{+}-ATPase in the E2·MgF_4^{2-}·$2K^{+}$ crystals. (**a**) The "average" conformation (PDB ID: 2ZXE); (**b**) thermally "opened" conformation as deduced by TLSMD[1]; (**c**) ouabain bound form (PDB ID: 3A3Y). Side chains of residues on M4E and those facing M4E are in space fill. Purple spheres (I and II) represent bound K^{+}. Stereo views approximately parallel to the membrane. Asterisks in **a** and **b** refer to Ile325, the side chain of which undergoes a conformation change in binding of ouabain (**c**).

Supplementary Table 1. Data collection and refinement statistics

	Tl^+ (0.75 min)	Tl^+ (1.5 min)	Tl^+ (3.5 min)
**Data collection**			
Cell dimensions[§]			
a, *b*, *c* (Å)	223.9, 51.0, 164.0	223.1, 50.8, 163.9	221.9, 50.9, 164.2
α, β, γ (°)	90, 105.0, 90	90, 104.7, 90	90, 104.5, 90
Resolution (Å)	50-2.6 (2.69-2.60)[*]	50-2.6 (2.69-2.60)	50-2.9 (3.00-2.90)
R_{sym}	0.064 (0.503)[*]	0.072 (0.656)	0.105 (0.634)
I/σ_I	20.1 (2.4)[*]	28.9 (2.6)	15.4 (1.8)
Completeness (%)	91.7 (86.9)[*]	99.3 (97.5)	96.5 (89.5)
Redundancy	3.0 (2.5)[*]	5.2 (3.9)	3.4 (2.4)
**Refinement**			
Resolution (Å)	15.0-2.6 (2.64-2.60)[*]	15.0-2.7 (2.74–2.70)	15.0-2.9 (2.96–2.90)
No. reflections	40,234 (897)	39,865 (878)	28,025 (580)
$R_{work/}$ R_{free}[†]	0.260 / 0.271 (0.383 / 0.494)[*]	0.265 / 0.281 (0.357 / 0.334)	0.288 / 0.294 (0.435 / 0.549)
Anomalous occupancy for Tl^+ (site I / II / C)	0.40 / 0.58 / 0.57	0.70 / 0.97 / 0.84	0.82 / 1.00 / 0.79
PDB accession code	5AVQ	5AVR	5AVS

	Tl^+ (5.0 min)	Tl^+ (7.0 min)	Tl^+ (8.5 min)
**Data collection**			
Cell dimensions			
a, *b*, *c* (Å)	221.5, 50.9, 164.1	222.5, 50.9, 163.9	221.0, 50.8, 163.4
α, β, γ (°)	90, 104.5, 90	90, 104.7, 90	90, 104.0, 90
Resolution (Å)	50-2.9 (2.99-2.90)	50-2.55 (2.62-2.55)	50-2.9 (3.00-2.90)
R_{sym}	0.077 (0.502)	0.071 (0.611)	0.069 (0.511)
I/σ_I	17.2 (2.2)	23.3 (2.4)	20.1 (2.1)
Completeness (%)	95.0 (86.5)	96.4 (85.9)	96.4 (85.6)
Redundancy	3.1 (2.3)	3.6 (2.9)	3.3 (2.5)
**Refinement**			
Resolution (Å)	15.0-2.9 (2.96-2.90)	15.0-2.55 (2.58–2.55)	15.0-2.9 (2.96–2.90)
No. reflections	27,810 (603)	42,045 (586)	28,635 (600)
$R_{work/}$ R_{free}[†]	0.271 / 0.262 (0.380 / 0.463)	0.266 / 0.269 (0.411 / 0.354)	0.286 / 0.300 (0.434 / 0.581)
Anomalous occupancy for Tl^+ (site I / II / C)	0.85 / 1.27 / 0.76	0.88 / 1.28 / 0.78	1.12 / 1.27 / 0.66
PDB accession code	5AVT	5AVU	5AVV

	Tl^+ (16.5 min)	Tl^+ (20.0 min)	Tl^+ (20.0 min)
**Data collection**			
Cell dimensions			
a, *b*, *c* (Å)	222.5, 50.9, 163.8	221.1, 50.8, 163.4	220.6, 50.7, 163.9
α, β, γ (°)	90, 104.7, 90	90, 103.7, 90	90, 103.6, 90
Resolution (Å)	50-2.6 (2.67-2.60)	50-3.3 (3.41-3.30)	50-3.45 (3.57-3.45)
R_{sym}	0.075 (0.581)	0.106 (0.637)	0.118 (0.755)
I/σ_I	22.9 (2.4)	16.0 (1.9)	14.7 (2.0)
Completeness (%)	97.9 (93.2)	84.3 (83.0)	99.7 (99.5)
Redundancy	4.1 (3.4)	3.3 (3.0)	3.7 (3.4)
**Refinement**			
Resolution (Å)	15.0-2.6 (2.64-2.60)	15.0-3.3 (3.41–3.30)	15.0-3.45 (3.55–3.45)
No. reflections	40,610 (784)	18,946 (1,118)	20,165 (1,166)
$R_{work/}$ R_{free} †	0.270 / 0.263 (0.406 / 0.486)	0.323 / 0.327 (0.545 / 0.605)	0.319 / 0.316 (0.494 / 0.459)
Anomalous occupancy for Tl^+ (site I / II / C)	1.12 / 1.19 / 0.81	1.22 / 1.12 / 0.48	1.14 / 1.21 / 0.52
PDB accession code	5AVW	5AVX	5AVY

	Tl^+ (55.0 min)	Tl^+ (55.0 min)	Tl^+ (85.0 min)
**Data collection**			
Cell dimensions			
a, *b*, *c* (Å)	222.7, 50.9, 164.7	224.2, 51.0, 164.5	222.0, 50.9, 164.0
α, β, γ (°)	90, 104.5, 90	90, 104.8, 90	90, 104.0, 90
Resolution (Å)	50-3.2 (3.30-3.20)	50-3.3 (3.41-3.30)	50-3.35 (3.46-3.35)
R_{sym}	0.115 (0.768)	0.104 (0.657)	0.120 (0.706)
I/σ_I	18.5 (2.1)	18.6 (2.2)	17.3 (2.1)
Completeness (%)	99.7 (99.2)	99.1 (98.1)	99.4 (98.7)
Redundancy	4.9 (4.3)	3.6 (3.2)	3.5 (3.1)
**Refinement**			
Resolution (Å)	15.0-3.2 (3.27-3.20)	15.0-3.3 (3.38–3.30)	15.0-3.35 (3.44–3.35)
No. reflections	26,622 (1,195)	24,209 (1,227)	22,611 (1,208)
$R_{work/}$ R_{free} †	0.305 / 0.309 (0.517 / 0.490)	0.290 / 0.291 (0.454 / 0.443)	0.304 / 0.318 (0.482 / 0.533)
Anomalous occupancy for Tl^+ (site I / II / C)	1.14 / 1.22 / 0.52	1.06 / 1.14 / 0.65	0.98 / 1.11 / 0.66
PDB accession code	5AVZ	5AW0	5AW1

	Tl^+ (85.0 min)	Tl^+ (100.0 min)
**Data collection**		
Cell dimensions		
a, *b*, *c* (Å)	221.3, 50.6, 163.5	222.2, 50.9, 164.3
α, β, γ (°)	90, 104.3, 90	90, 104.2, 90
Resolution (Å)	50-3.2 (3.29-3.20)	50-3.35 (3.46-3.35)
R_{sym}	0.110 (0.703)	0.115 (0.747)
I/σ_I	17.9 (1.9)	14.2 (2.1)
Completeness (%)	99.4 (99.4)	99.3 (98.9)
Redundancy	3.8 (3.6)	3.6 (3.2)
**Refinement**		
Resolution (Å)	15.0-3.2 (3.28-3.20)	15.0-3.35 (3.44–3.35)
No. reflections	25,444 (1,193)	22,253 (1,231)
$R_{work/}$ R_{free} †	0.295 / 0.289 (0.436 / 0.517)	0.312 / 0.311 (0.527 / 0.477)
Anomalous occupancy for Tl^+ (site I / II / C)	1.11 / 1.12 / 0.61	1.13 / 1.20 / 0.59
PDB accession code	5AW2	5AW3

	Rb^+ (1.5 min)	Rb^+ (2.2 min)	Rb^+ (5.5 min)
**Data collection**			
Cell dimensions			
a, *b*, *c* (Å)	219.8, 50.6, 162.5	219.5, 50.5, 162.6	220.6, 50.6, 163.2
α, β, γ (°)	90, 104. 4 , 90	90, 104.1, 90	90, 104.6, 90
Resolution (Å)	50-2.7 (2.78-2.70)	50-2.9 (2.99-2.90)	50-2.8 (2.88-2.80)
R_{sym}	0.081 (0.739)	0.113 (0.847)	0.092 (0.774)
I/σ_I	19.6 (1.9)	15.8 (1.8)	18.7 (2.0)
Completeness (%)	96.6 (90.7)	96.4 (90.2)	96.3 (87.7)
Redundancy	2.8 (2.2)	3.6 (2.7)	3.4 (2.6)
**Refinement**			
Resolution (Å)	15.0-2.8 (2.86-2.80)	15.0-2.9 (2.97–2.90)	15.0-2.8 (2.86–2.80)
No. reflections	29,427 (685)	25,316 (572)	29,630 (622)
$R_{work/}$ R_{free} †	0.285 / 0.275 (0.523 / 0.516)	0.303 / 0.315 (0.553 / 0.481)	0.282 / 0.288 (0.504 / 0.567)
Anomalous occupancy for Rb^+ (site I / II / C)	0.40 / 0.51 / 0.55	0.69 / 0.86 / 0.74	0.76 / 0.95 / 0.73
PDB accession code	5AW4	5AW5	5AW6

	Rb^+ (11.3 min)	Rb^+ (co-crystal)	Native[‡]
**Data collection**			
Cell dimensions			
a, b, c (Å)	219.2, 50.5, 162.6	221.1, 50.7, 163.3	220.1, 50.7, 163.1
α, β, γ (°)	90, 104.3, 90	90, 104.9, 90	90, 104.5, 90
Resolution (Å)	50-2.8 (2.88-2.80)	50-2.8 (2.86-2.80)	50-2.8 (2.89-2.80)
R_{sym}	0.098 (0.858)	0.083 (0.582)	0.059 (0.517)
I/σ_I	17.0 (1.9)	19.5 (1.7)	21.0 (1.8)
Completeness (%)	92.6 (82.3)	97.2 (79.9)	93.6 (67.6)
Redundancy	3.5 (2.8)	5.7 (4.3)	3.5 (3.0)
**Refinement**			
Resolution (Å)	15.0-2.9 (2.97-2.90)	15.0-2.8 (2.86–2.80)	15-2.8 (2.85-2.80)
No. reflections	26,660 (790)	30,700 (548)	39,354 (578)
$R_{work/}$ R_{free}[†]	0.290 / 0.296 (0.500 / 0.509)	0.259 / 0.262 (0.304 / 0.293)	0.245 / 0.255 (0.254 / 0.255)
Anomalous occupancy for Rb^+ (site I / II / C)	0.77 / 1.03 / 0.91	1.00 / 1.00 / 1.00	0.00 / 0.00 / 0.00
PDB accession code	5AW7	5AW8	5AW9

*Values for the highest resolution shell are provided in parentheses.

[§]Space group and unit cell dimensions for native crystal (PDB ID: 2ZXE) are $C2$, and a = 223.8, b = 50.9, c = 163.8 (Å), α = 90º, β = 105.1º and γ = 90º.

[†]R values obtained by rigid body refinement using 2ZXE as the reference molecule and treating the entire protein as one segment.

[‡]Native crystal used for $|F_{obs}(Rb^+)|-|F_{obs}(K^+)|$ difference Fourier maps of Rb^+-substituted crystals.

References

1 Painter, J. & Merritt, E. A. Optimal description of a protein structure in terms of multiple groups undergoing TLS motion. *Acta Crystallogr. D* **62**, 439-450 (2006).

2. Eyal, E., Lum, G., Bahar, I. The anisotropic network model web server at 2015 (ANM 2.0) *Bioinformatics* **31**, 1487-1489 (2015)

3. K. Suhre & Y.H. Sanejouand, ElNémo: a normal mode web-server for protein movement analysis and the generation of templates for molecular replacement. *Nucleic Acids Res.* **32**, W610-W614 (2004).
